# Mouse offspring conceived by in vitro fertilization exhibit accelerated reproductive aging through premature ovarian insufficiency

**DOI:** 10.1172/JCI201633

**Published:** 2026-06-30

**Authors:** Eric A. Rhon-Calderon, Cassidy N. Hemphill, Alexandra J. Savage, Ana Domingo-Muelas, Zhengfeng Liu, Christopher J. Krapp, Laren Riesche, Nicolas Plachta, Richard M. Schultz, Marisa S. Bartolomei

**Affiliations:** 1 Epigenetics Institute, Department of Cell and Developmental Biology, Perelman School of Medicine,; 2 Department of Cell and Developmental Biology, and; 3 Department of Biology, School of Arts and Sciences, University of Pennsylvania, Philadelphia, Pennsylvania, USA.; 4 Department of Microbiology and Molecular Genetics, University of California, Davis, California, USA.; 5 Center for Women’s Health and Reproductive Medicine, Philadelphia, Pennsylvania, USA.

**Keywords:** Development, Reproductive biology, Embryonic development, Epigenetics, Transcriptomics

## Abstract

Reproductive aging is characterized by a progressive decline of reproductive function, with broad implications for overall health and longevity. Environmental factors, including assisted reproductive technologies (ARTs), can accelerate reproductive aging by promoting premature ovarian insufficiency in females. In vitro fertilization (IVF), though widely used and generally considered safe, has been associated with lasting effects on offspring health. Using a mouse model that closely approximates human IVF, we demonstrated that IVF accelerated reproductive aging in female offspring by inducing premature ovarian insufficiency. IVF-conceived female mice exhibited altered ovarian function, reduced follicle reserve, disrupted endocrine profiles, and transcriptomic and epigenetic changes consistent with premature reproductive decline. These findings reveal long-term consequences of IVF on female reproductive health and highlight the need to understand how early-life interventions influence reproductive longevity.

## Abstract 2

IVF conception accelerates reproductive aging in female offspring, resulting in ovarian dysfunction, follicle loss, altered gene regulation, epigenetic dysregulation, and impaired fertility.

## Graphical abstract

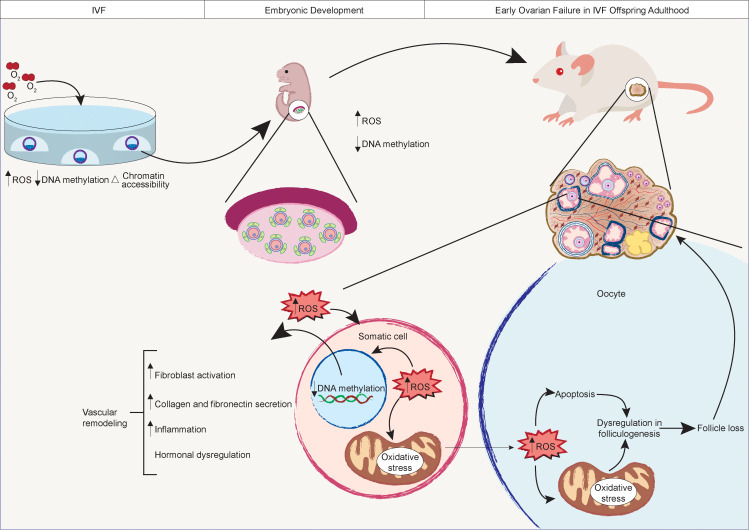

## Introduction

Reproductive aging manifests as a physiological decline of biological functions essential for reproduction and overall health (1). In females, reproductive aging involves the gradual decline in egg quality and quantity, accompanied by a decrease in reproductive hormones, e.g., estradiol, ultimately leading to menopause in humans or reproductive senescence in rodents (1). In some women, reproductive aging occurs prematurely, manifesting as primary ovarian insufficiency (POI), a condition characterized by the loss of ovarian function before the age of 40 (2). POI is an extreme example of premature reproductive aging, which encompasses earlier and accelerated declines in ovarian reserve and endocrine function that negatively affect overall health and fertility and is driven by genetic, environmental, and epigenetic factors (3). Adverse conditions during early development, including those associated with assisted reproductive technologies (ARTs), could contribute to premature reproductive aging by disrupting epigenetic programming and metabolic processes (4–6). These alterations can impair cellular function, increase mitochondrial stress, and lead to the early onset of age-related conditions, including reduced reproductive functions.

ARTs involve noncoital methods of conception that are used to treat infertility (7). Since the first successful human birth from in vitro fertilization (IVF) in 1978 (8), IVF and intracytoplasmic sperm injection have become the most widely used technologies to successfully achieve fertilization in vitro (8). In both humans and mice, ART procedures are generally considered safe, but recent studies have shown that ART pregnancies are associated with higher risks of perinatal, neonatal, and placental adverse outcomes, rare genetic syndromes, and abnormal male offspring reproductive outcomes (9).

IVF-associated adverse outcomes may be linked to critical developmental windows of gamete and preimplantation embryo epigenetic reprogramming, which are highly susceptible to external stresses (9, 10) and strongly influenced by the duration and conditions of the embryo culture (11). Despite ongoing refinements in IVF procedures to enhance fertilization and pregnancy rates, improvements in offspring outcomes have not been successful. The increasing use of IVF, driven by delayed childbearing, same-sex couples seeking parenthood, and rising infertility rates, underscores the need for further study on potential adverse outcomes for future generations (12). Understanding the risk of premature reproductive aging in IVF-conceived individuals is crucial for evaluating long-term health risks and refining assisted reproductive practices.

In humans, female offspring conceived through IVF are generally healthy, but recent research has identified adverse changes in cardiovascular and metabolic parameters (13). Additionally, small cohort studies involving adolescent females have reported a reduced ovarian reserve, altered timing of puberty, and hormonal imbalances (14). Despite these findings, the overall effect of IVF on the female reproductive system remains unknown, and the mechanisms driving these adverse outcomes are yet to be fully elucidated.

Mice have become essential models for studying human disease and reproductive biology and have been instrumental in the development and refinement of ARTs (15). Mouse studies have shown that IVF is associated with increased risks of metabolic syndrome in offspring, including altered glucose, insulin, triglyceride, and cholesterol levels, as well as changes in liver transcriptomes and proteomes compared with naturally conceived offspring (4–6, 16–21). Furthermore, our laboratory has shown that IVF disrupts the reproductive system in male offspring, raising concerns about female reproductive effects (22).

Despite observed adverse outcomes in multiple fetal and adult organs of IVF-conceived mice (4, 16, 23, 24), no study to date has focused on the female reproductive system and the risk of premature aging. To address this gap, we examined the morphological and molecular effects of IVF on the ovaries and oocytes of female offspring and evaluated whether these alterations are associated with an increased risk of premature reproductive aging. Our findings reveal that IVF-conceived female mice showed changes consistent with accelerated reproductive aging.

## Results

### Reduced estrogen levels and altered DNA methylation are associated with premature reproductive aging in IVF-conceived offspring.

**Figure 1:**
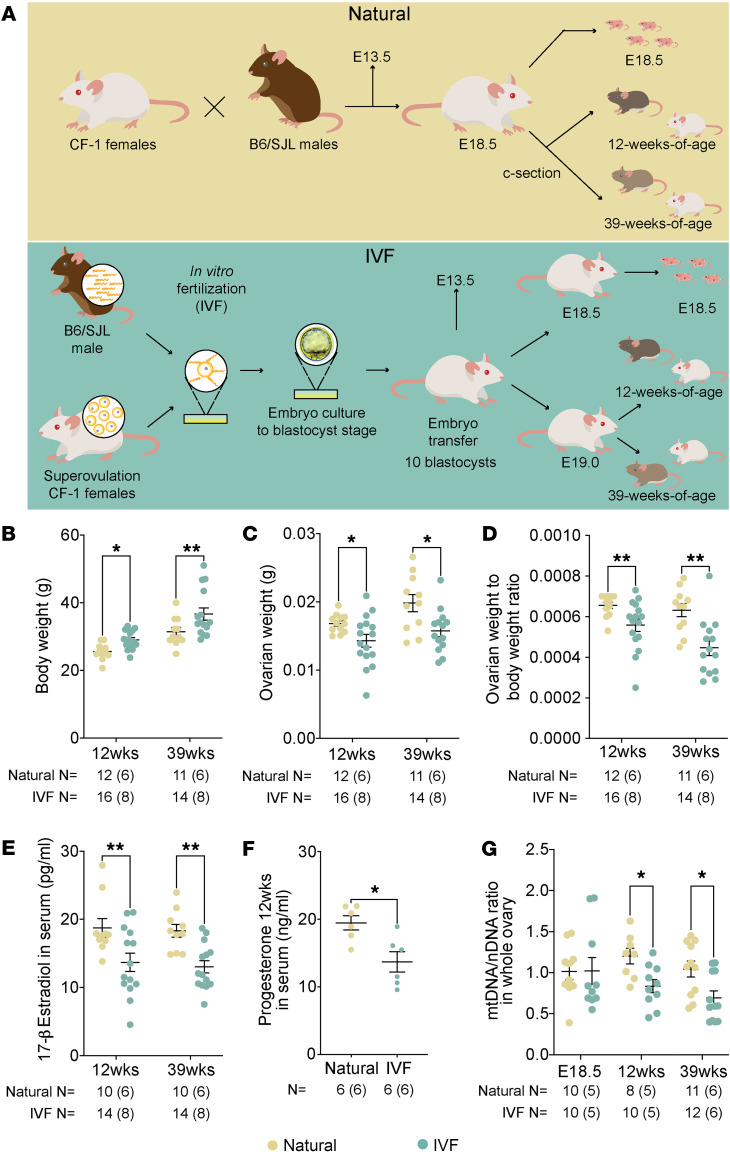
Mouse model and general parameters. (**A**) Mouse model: Naturally conceived embryos (Natural) were generated using B6/SJL male mice and CF-1 female mice, and IVF embryos were produced using capacitated sperm from B6/SJL mice and eggs from superovulated CF-1 mice. After embryo culture to the blastocyst stage, 10 blastocysts were transferred to pseudopregnant females. At E13.5, indicated by the arrow, pregnant females from both groups were euthanized, fetuses were collected by c-section, and fetal gonads were dissected for molecular analysis. At E18.5, pregnant females from both groups were c-sectioned, and fetuses were delivered and collected for molecular analysis. For adult cohorts, c-section–delivered pups at E18.5 (natural) or E19.0 (IVF) were fostered with WT dams and monitored until 12 or 39 weeks of age. Shown are (**B**) body weights before 6 hours fasting; (**C**) ovarian weights; (**D**) ovary to body weight ratios; (**E**) serum estradiol levels; and (**F**) progesterone levels assayed by ELISA. (**G**) The ratio of mtDNA relative to nDNA measured in whole ovarian DNA by RT-qPCR is depicted for all time points (see Methods for primers). Data are presented as the mean ± SEM, and sample sizes are indicated below each panel as the number of animals (litters). The black line represents the mean of each group. Normality was assessed using the Shapiro-Wilk test. Data that did not meet the normality assumption were analyzed using a nonparametric Mann-Whitney *U* test, whereas normally distributed data were analyzed using an unpaired 2-tailed *t* test with Welch’s correction. *P < 0.05 and **P < 0.01, for IVF groups compared with Naturals.

To investigate reproductive function in female IVF offspring, we examined ovaries using our mouse model (Figure 1A). At 12 and 39 weeks, body and ovarian weights were significantly higher and lower, respectively, in IVF-conceived females compared with naturally conceived controls (referred to hereafter as Natural), resulting in a reduced ovarian-to-body weight ratio (Figure 1, B–D). The reduced ovarian weight could adversely affect ovarian function because ovaries are the primary producers of several key female sex hormones, including estradiol and progesterone (25). Consistent with reduced ovarian weight, IVF female mice had lower serum estradiol levels at both 12 and 39 weeks and reduced progesterone levels at 12 weeks (Figure 1, E and F). Notably, in mice, estradiol and progesterone are important indicators of ovarian endocrine function and contribute to the regulation of follicular development and the reproductive tract environment (26); disruptions in estradiol levels can impair oocyte quality, whereas changes in progesterone are associated with estrous cycle alterations, uterine remodeling, and future pregnancy complications (27).

Changes in mitochondrial function are considered a hallmark of aging (28), in which both mitochondrial DNA (mtDNA) copy number relative to nuclear DNA (nDNA) and mitochondrial function decline with age in various tissues, including the ovaries (28). To determine whether female IVF offspring exhibit mtDNA changes, we performed real-time quantitative PCR (RT-qPCR) using a mtDNA marker normalized to nDNA. While no significant differences were observed at E18.5, we detected a significant decrease in the mtDNA/nDNA ratio at both 12 and 39 weeks (Figure 1G), suggesting the onset of an aging phenotype in IVF female offspring.

Collectively, these findings suggest that IVF contributed to premature ovarian aging by reducing estrogen levels and mtDNA content, each of which has been linked to ovarian dysfunction, mitochondrial impairment, and premature aging.

### Altered ovarian morphology is associated with premature reproductive aging in IVF offspring.

**Figure 2:**
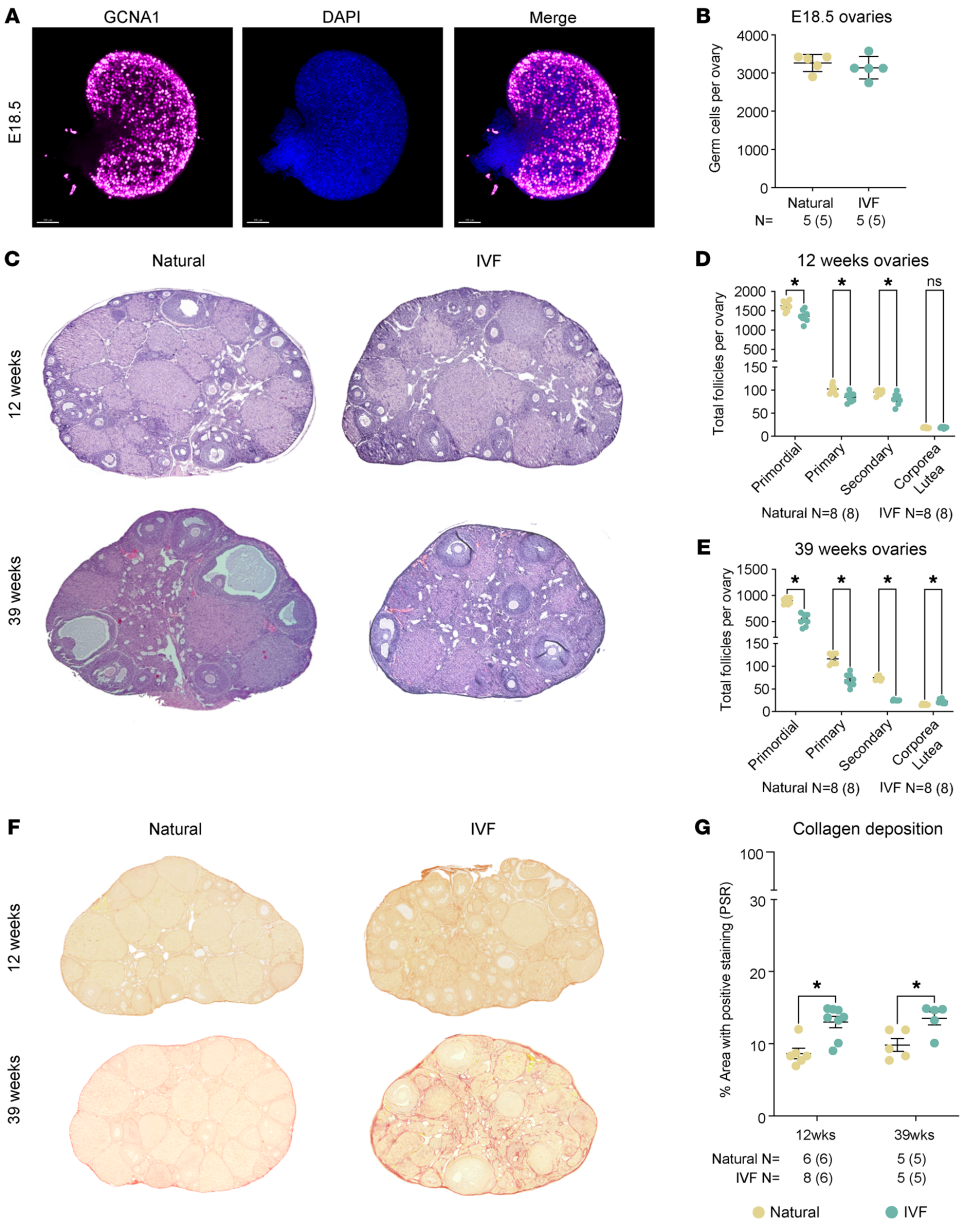
Ovarian morphology analysis. (**A**) E18.5 natural whole ovarian staining for GCNA1 (germ cells) and DAPI (nuclei). Scale bars: 100 μm. (**B**) Quantification of whole ovarian staining at E18.5. (**C**) Ovarian cross-sections stained with H&E at 12 and 39 weeks. Original magnification, ×40. (**D**) Total follicle number per ovary at 12 weeks. (**E**) Total follicle number per ovary at 39 weeks. (**F**) Ovarian cross-sections stained with PS at 12 and 39 weeks. Original magnification, ×40. (**G**) Quantification of collagen deposition shown as the percentage of PSR^+^ area. Each data point represents one biological replicate. Sample sizes are indicated below each panel as the number of animals (litters). Data are presented as the mean ± SEM. Normality was assessed using the Shapiro-Wilk test. Data that did not meet the normality assumption were analyzed using the nonparametric Mann-Whitney *U* test, whereas normally distributed data were analyzed using an unpaired 2-tailed *t* test with Welch’s correction. *P < 0.05 compared with Naturals.

Oocyte maturation and ovulation are tightly coordinated processes that require bidirectional communication between the oocyte and surrounding follicular cells; disruption of this communication can impair meiotic maturation, cumulus expansion, follicle rupture, and release of a developmentally competent oocyte (29). Furthermore, early depletion of ovarian follicles is associated with reductions in ovarian size and estradiol production (30). Thus, adverse effects on the ovarian reserve or ovarian function may compromise folliculogenesis, ultimately affecting female fertility. To determine whether IVF affects the establishment or postnatal maintenance of the ovarian reserve, we next examined germ cell numbers during fetal development and follicle populations in adult ovaries. First, we quantified germ cells at E18.5 using whole-ovary staining for germ cell nuclear antigen 1 (GCNA1) (TRA98), a germ cell marker (31), and observed no difference between IVF and naturally conceived ovaries (Figure 2, A and B). In contrast, H&E staining of whole ovaries revealed fewer primordial, primary, and secondary follicles at both 12 and 39 weeks (Figure 2, C–E). The absence of differences in germ cell numbers at E18.5, together with reduced follicle numbers postnatally, suggests that accelerated germ cell depletion occurs after birth and could potentially be associated with transcriptional alterations in the ovarian somatic cells involved in follicle activation and development.

Collagen is a key structural component of the ovary that supports follicle development and oocyte maturation, but excessive deposition with age leads to fibrosis and impaired ovarian function (32, 33). To assess whether IVF influences collagen accumulation, we performed Picrosirius red (PR) staining and observed increased collagen density in IVF ovaries at both 12 and 39 weeks compared with controls (Figure 2, F and G).

Taken together, these results indicate that IVF induced significant alterations in ovarian morphology, including a reduced primordial follicle pool, and increased collagen deposition, consistent with features of primary ovarian insufficiency.

### Ovarian transcriptome in IVF offspring reveals genes linked to premature reproductive aging.

**Figure 3:**
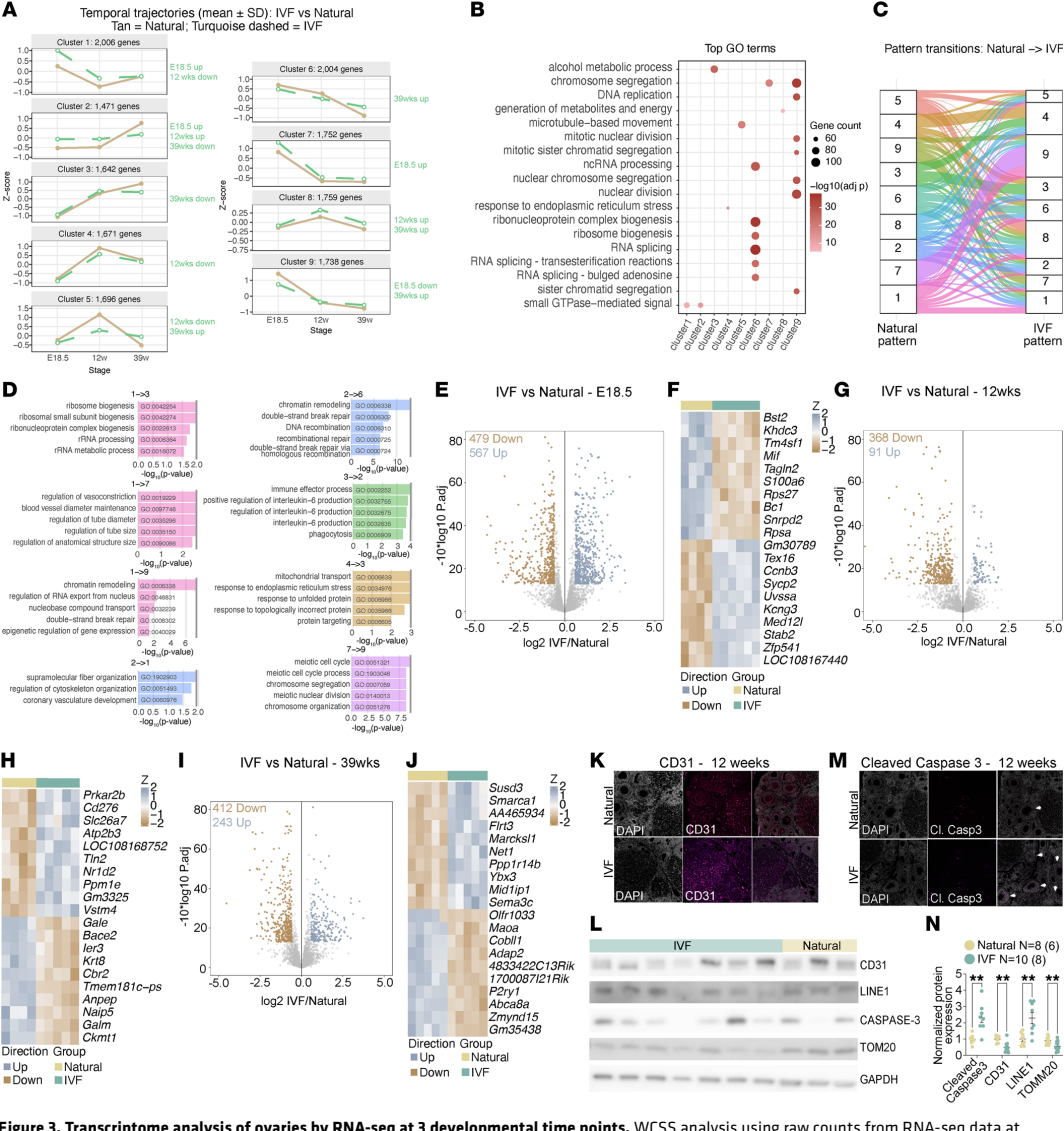
Transcriptome analysis of ovaries by RNA-seq at 3 developmental time points. WCSS analysis using raw counts from RNA-seq data at E18.5, 12 weeks, and 39 weeks. (**A**) Nine temporal expression patterns (*k* = 9) across mouse ovarian development, using Natural as the training group. (**B**) Top 20 enriched biological processes associated with ovarian function and their respective temporal expression cluster. (**C**) Changes in temporal expression patterns for all genes from Natural to IVF groups. (**D**) Top 5 enriched biological processes using the genes that change temporal expression patterns (clusters). (**E**–**J**) Differential expression analysis was performed using DeSeq2. (**E**) Volcano plot (E18.5 gonads). (**F**) Heatmap with the most DEGs upregulated (Up) or downregulated (Down) and (**G**) corresponding volcano plot (12-week ovaries). (**H**) Heatmap with the most DEGs up- or downregulated and (**I**) corresponding volcano plot (39-week ovaries). (**J**) Heatmap with the most DEGs up- or downregulated. Color boxes at the top denote the experimental group. (**K**) Immunofluorescence staining for CD31 in 12-week ovaries. Original magnification, ×400. (**L**) Representative Western blot for CD31, LINE1 ORF1p, cleaved caspase 3, TOM20, and GAPDH. (**M**) Immunofluorescence staining for cleaved caspase 3 expression in 12-week ovaries. (**N**) Quantification of normalized protein expression for cleaved caspase 3, CD31, LINE1 ORF1p, and TOM20 in 12-week ovaries. Western blot band intensities were normalized to GAPDH and subsequently normalized within each blot to the mean intensity of the Natural group to account for inter-blot variability. Each data point represents 1 biological replicate. Sample sizes are indicated above each panel as the number of animals (litters). Data are presented as the mean ± SEM. Normality was assessed using the Shapiro-Wilk test. Data that did not meet the normality assumption were analyzed using the nonparametric Mann-Whitney *U* test, whereas normally distributed data were analyzed using an unpaired 2-tailed *t* test with Welch’s correction. *P < 0.05 and *P < 0.01 compared with Naturals.

To determine whether the phenotypic changes described above were associated with altered gene expression and age-related patterns in IVF-conceived females, we performed RNA-seq on whole ovaries collected at E18.5, 12 weeks, and 39 weeks. Principal component analysis (PCA) revealed clear clustering differences between IVF- and naturally conceived groups (Supplemental Figure 1, A–C; supplemental material available online with this article; https://doi.org/10.1172/JCI201633DS1). The observed variability suggests that IVF-associated transcriptional remodeling was heterogeneous rather than uniformly present across all individuals. Accordingly, we used temporal clustering analysis to identify coordinated age-related gene expression trajectories and determine whether IVF alters these developmental transcriptional programs. To define age-dependent gene expression dynamics across ovarian development and aging, we applied within-cluster sum of squares (WCSS) analysis to determine the optimal number of transcriptional trajectories represented in the dataset (34). We used WCSS to evaluate how genes grouped according to similarities in their expression patterns across the 3 developmental time points analyzed (E18.5, 12 weeks, and 39 weeks), allowing identification of coordinated temporal programs rather than isolated differentially expressed genes (DEGs). This approach detected biologically meaningful clusters reflecting development-, reproductive maturity–, and aging-associated transcriptional transitions in the ovary. On the basis of the WCSS inflection point, we identified 9 temporal clusters spanning E18.5 to 39 weeks (Figure 3A).

In naturally conceived female mice, genes followed coordinated age-dependent trajectories, with subsets either increasing or decreasing across ovarian development and aging. In contrast, IVF mouse ovaries showed disrupted trajectories across multiple clusters, indicating that IVF altered the normal timing of ovarian transcriptional programs (Figure 3A). Gene ontology (GO) enrichment analysis showed that these altered temporal programs converged on pathways directly relevant to POI, including mitochondrial metabolism, autophagy, cell-cycle regulation, chromosome segregation, RNA processing, and tissue homeostasis (Figure 3B and Supplemental Table 1) (35, 36). These processes are essential for maintaining follicle survival, oocyte quality, and ovarian reserve. Importantly, genes that shifted cluster identity between Natural and IVF ovaries were enriched for pathways involved in metabolism, chromatid maintenance, chromosome integrity, cell-cycle regulation, and respiration (Figure 3, C and D). Thus, IVF did not simply alter individual genes, but disrupted coordinated transcriptional programs that maintain genome stability, mitochondrial function, and follicular homeostasis, core mechanisms implicated in reproductive aging and POI.

We next performed differential expression analysis and identified 567 upregulated and 479 downregulated genes at E18.5 (Figure 3E and Supplemental Table 2), 91 upregulated and 368 downregulated genes at 12 weeks (Figure 3G and Supplemental Table 3), and 243 upregulated and 412 downregulated genes at 39 weeks (Figure 3I and Supplemental Table 4). We observed the largest number of DEGs at E18.5, and this difference did not appear to worsen with age. Several DEGs are directly linked to ovarian health and POI. *Hfm1* and *Spata22*, both required for homologous recombination and meiotic progression, were dysregulated in our study and are associated with impaired oocyte quality and follicle loss (37). We found that *Star*, which mediates cholesterol transport for estrogen biosynthesis, was altered in a manner consistent with the reduced estradiol levels detected in IVF-conceived female mice (38). *Esr1*, encoding the estrogen receptor, was downregulated, potentially driving impaired follicle development and ovulation (39). Additionally, we observed that *Gdf9*, a critical oocyte-derived growth factor for granulosa cell proliferation and folliculogenesis, was reduced, in line with the lower follicle counts (40). Importantly, the dysregulation of genes involved in steroidogenesis and hormone signaling provides a molecular basis for the reduced estradiol and progesterone levels observed in IVF offspring.

GO enrichment analysis of DEGs revealed pathways related to metabolism, mitochondrial function, cell-cycle regulation, and gamete maturation at E18.5; metabolism and cell-cycle regulation at 12 weeks; and metabolism and follicle development at 39 weeks (Supplemental Figure 1, E–G), highlighting progressive metabolic and developmental dysregulation across ovarian maturation stages. We next asked whether the identified DEGs were associated with established hallmarks of aging (41). Pathway enrichment analysis revealed that genes dysregulated in IVF ovaries were significantly associated with multiple aging-related processes (Supplemental Figure 1, H and I). At 12 weeks, DEGs were enriched for pathways linked to altered intercellular communication, chronic inflammation, deregulated nutrient sensing, and genomic instability. By 39 weeks, these enrichments expanded to include mitochondrial dysfunction, loss of proteostasis, and stem cell exhaustion, hallmarks that together reflect progressive cellular decline.

We further identified the most upregulated and downregulated DEGs at each age (Figure 3, F, H, and J), many of which play roles in ovarian homeostasis. Notably, intersection of the DEG datasets across all time points revealed 2 consistently altered genes — *Cdh13* and *Slc2a1* (Supplemental Figure 1, J and K) — highlighting them as potential key mediators of IVF-associated ovarian dysfunction and accelerated reproductive aging. *Cdh13*, a cell adhesion molecule, has been associated with ovarian syndromes and may contribute to altered ovarian morphology (42), while *Slc2a1* (also known as *Glut1*), a glucose transporter essential for granulosa-oocyte energy transfer (43), was consistently affected, providing a mechanistic link to the observed disrupted ovarian metabolism.

Because the RNA-seq analysis identified pathways related to apoptosis, vascular remodeling, transposable element regulation, and mitochondrial function, we next performed immunofluorescence and Western blot analyses to validate these molecular signatures at the protein and tissue levels. Ovarian aging and POI are associated with increased follicular atresia, apoptosis, and reduced microvessel density (44, 45). Therefore, we assessed expression levels of cleaved caspase 3, a marker of apoptosis, and CD31, an endothelial cell adhesion molecule commonly used to evaluate vascular density and microvascular integrity, in ovaries of 12-week-old mice. Consistent with the RNA-seq signatures, IVF ovaries showed increased cleaved caspase 3 and reduced CD31 immunofluorescence, suggesting increased apoptotic activity and impaired ovarian vascularization (Figure 3, K and L). Immunoblotting further validated altered protein levels of cleaved caspase 3, CD31, long interspersed nuclear element-1 (LINE1), open reading frame 1 protein (ORF1p), and translocase of outer mitochondrial membrane 20 (TOM20) in IVF ovaries (Figure 3, M and N), supporting the transcriptomic evidence of altered apoptosis, vascular integrity, transposable element regulation, and mitochondrial function in IVF-conceived female offspring. Increased LINE1 activity has been linked to elevated dsDNA breaks, mitochondrial dysfunction, and apoptosis (46), which is consistent with the reduced mtDNA levels and increased cleaved caspase 3 observed in our model.

Taken together, these results align with the IVF-associated ovarian phenotypes and indicate that, as early as 12 weeks, the ovary exhibits transcriptional signatures of aging that intensify with time, suggesting that IVF accelerates molecular pathways driving reproductive aging.

### Single oocyte transcriptome in IVF offspring reveals genes linked to premature reproductive aging.

**Figure 4:**
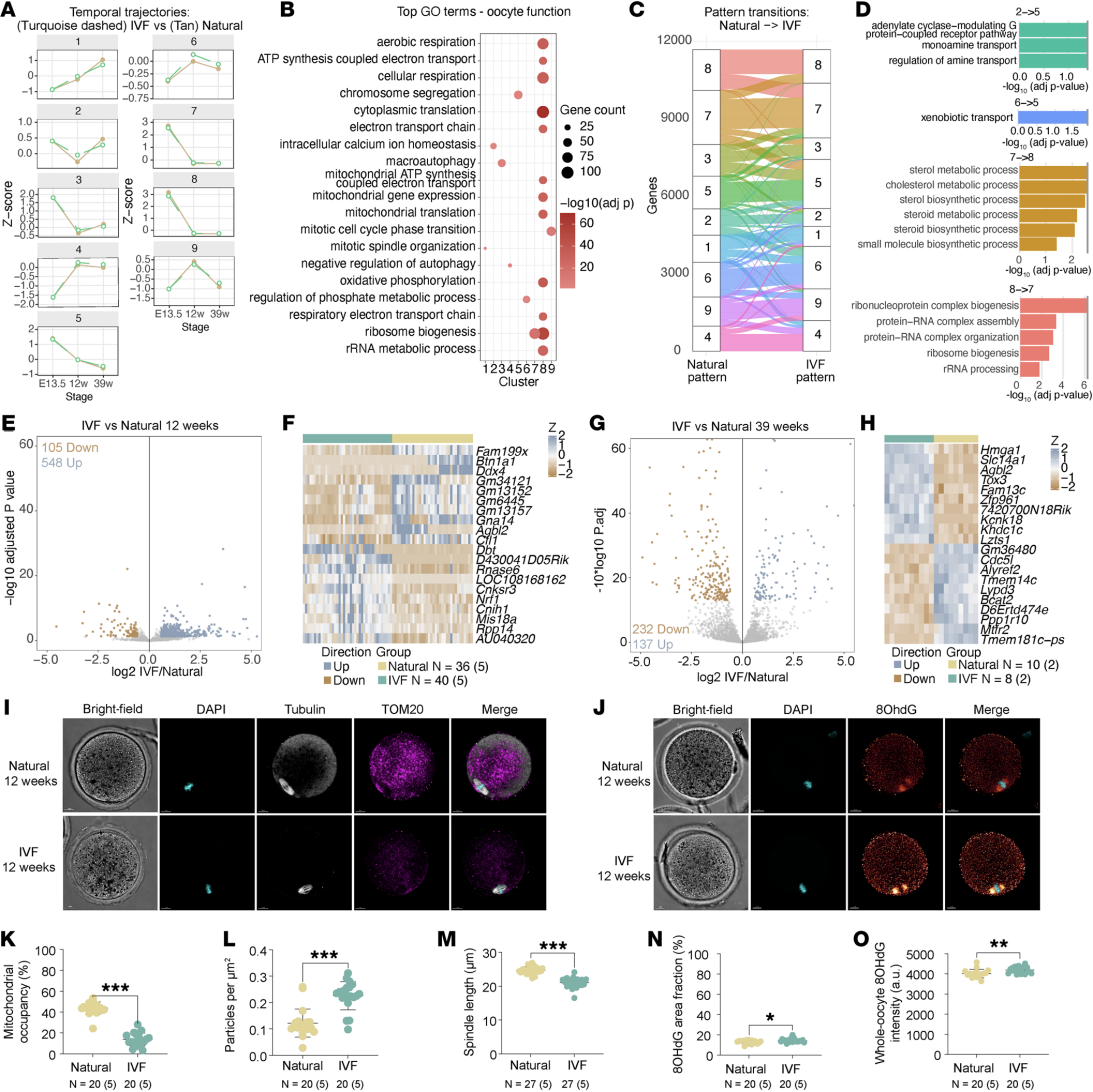
Transcriptomic analysis of primordial germ cells and single oocytes across development. (**A**) WCSS analysis and temporal expression clustering (*k* = 9) using normalized RNA-seq counts from E13.5 PGCs, 12-week oocytes, and 39-week oocytes, with Natural samples used as the training group. (**B**) Top enriched biological processes associated with temporal expression clusters. (**C**) Sankey plot showing shifts in temporal gene expression patterns between Natural and IVF groups. (**D**) Top enriched biological processes associated with genes displaying altered temporal expression dynamics. Differential expression analysis was performed using DESeq2. (**E**) Volcano plot of DEGs in 12-week oocytes, with significantly upregulated genes shown in blue and downregulated genes shown in orange. (**F**) Heatmap of the top upregulated and downregulated DEGs in 12-week oocytes. (**G**) Volcano plot of DEGs in 39-week oocytes. (**H**) Heatmap of the top upregulated and downregulated DEGs in 39-week oocytes. Heatmaps depict row-scaled normalized expression values, and color bars above heatmaps indicate the experimental group assignment. Differential expression significance was determined using DESeq2 with an adjusted *P* value of less than 0.05. (**I**) Representative 12-week oocytes stained for DAPI (cyan), α-tubulin (white), and TOM20 (magenta). Original magnification, ×100; scale bars: 10 μm. (**J**) Representative 12-week oocytes stained for DAPI (cyan) and 8-OHdG (magenta). Original magnification, ×100; scale bars: 10 μm.. (**K**) Quantification of mitochondrial occupancy within oocytes. (**L**) Quantification of TOM20^+^ particles per μm^2^. (**M**) Quantification of meiotic spindle length. (**N**) Quantification of the 8OHdG^+^ area fraction. (**O**) Quantification of whole oocyte 8OHdG fluorescence intensity. Each dot represents 1 biological replicate. Sample sizes are indicated below each panel as the number of oocytes (litters). Data are presented as the mean ± SEM. Normality was assessed using the Shapiro-Wilk test. Data that did not meet the normality assumption were analyzed using the nonparametric Mann-Whitney *U* test, whereas normally distributed data were analyzed using an unpaired 2-tailed *t* test with Welch’s correction. *P < 0.05, **P < 0.01, and ***P < 0.001 compared with Naturals. adj, adjusted.

Given the cellular complexity of the ovary, we focused on germ cells using a modified SMART-Seq (47) protocol that enabled sequencing of small numbers of primordial germ cells (PGCs), oocytes, or cumulus cells from E13.5, 12-week-old, and 39-week-old female mice. PCA of E13.5 PGC transcriptomes showed partial separation between Natural and IVF samples, but clustering was not complete, with some IVF and Natural samples overlapping in the PCA space. This observation suggests that IVF-associated transcriptional changes in germ cells are heterogeneous rather than uniformly present across all samples (Supplemental Figure 2B). To define age-dependent transcriptional dynamics in germ cells and oocytes, we applied WCSS analysis across E13.5, 12 weeks, and 39 weeks. This analysis identified 9 temporal expression clusters that captured gene expression trajectories across development and aging (Figure 4A). In Natural females, these clusters showed distinct developmental patterns, with subsets of genes decreasing or increasing from embryonic to adult stages. In contrast, IVF-conceived female mice showed altered trajectories across multiple clusters, indicating disruption of normal oocyte transcriptional aging programs.

To assess the biological relevance of these clusters, we performed GO enrichment analysis (Figure 4B and Supplemental Table 5). Several clusters were enriched for pathways essential for oocyte function, including aerobic respiration, ATP synthesis–coupled electron transport, cellular respiration, chromosome segregation, cytoplasmic translation, electron transport chain activity, mitochondrial gene expression, oxidative phosphorylation, ribosome biogenesis, and rRNA metabolic processes. These pathways are central to oocyte maturation, mitochondrial function, protein synthesis, stress defense, and genome stability, all of which are critical determinants of oocyte quality (48–50). Together, these clusters highlight coordinated transcriptional disturbances in translation, RNA metabolism, energy regulation, and stress defense that collectively drive ovarian aging in IVF-conceived female mice.

We next examined genes that shifted temporal cluster membership between Natural and IVF oocytes (Figure 4C). GO analysis of these pattern-switching genes revealed enrichment for pathways related to protein-coupled receptor signaling, monoamine transport, xenobiotic transport, sterol and cholesterol metabolism, steroid biosynthesis, ribonucleoprotein complex biogenesis, protein-RNA complex assembly, and rRNA processing (Figure 4D), which are important for oocyte maturation. These findings suggest that IVF alters coordinated transcriptional programs involved in metabolism, steroid regulation, RNA processing, and oocyte functional maturation (48) (Figure 4D).

Differential expression analysis revealed no significant DEGs at E13.5 (Supplemental Figure 2A). At 12 weeks, IVF oocytes showed 548 upregulated and 105 downregulated genes (Figure 4E and Supplemental Table 6), whereas at 39 weeks, IVF oocytes showed 137 upregulated and 232 downregulated genes (Figure 4G; Supplemental Table 7). Heatmaps of the top upregulated and downregulated genes at each time point showed distinct expression differences between Natural and IVF oocytes (Figure 4, F and H). Together, these data indicate that IVF-associated transcriptional differences become more evident postnatally and persist into reproductive aging.

GO enrichment analysis of DEGs revealed enrichment for pathways related to cell-cycle regulation and metabolism at both 12 and 39 weeks (Supplemental Figure 2, E and F), highlighting disruptions in processes essential for oocyte quality, follicular maintenance, and overall ovarian function. We next examined whether dysregulated genes in oocytes were associated with hallmarks of aging (Supplemental Figure 2, G and H). At 12 weeks, IVF oocytes showed enrichment for pathways linked to cellular senescence, deregulated nutrient sensing through TOR signaling, genomic instability, loss of proteostasis, and mitochondrial dysfunction, indicating early activation of aging-associated stress responses. By 39 weeks, these transcriptional signatures intensified, with additional enrichment for chronic inflammation, mitochondrial respiratory defects, and stem cell exhaustion, consistent with progressive energetic decline and reduced oocyte quality.

Intersection of single-oocyte DEGs from 12 and 39 weeks revealed 10 overlapping upregulated genes in IVF oocytes (Supplemental Figure 2I). Among these, *Il17f* and *Rfxank* are associated with inflammatory and immune-related pathways (51), *Cdc42ep3* and *Hps5* suggest cytoskeletal and vesicle/lysosomal remodeling (52), and *Ercc* may reflect a compensatory response to genomic stress (53). Together, these recurrently upregulated genes support activation of aging-associated inflammatory, immune, and stress-response pathways in IVF oocytes. In contrast, 11 genes were downregulated in IVF oocytes at both 12 and 39 weeks (Supplemental Figure 2J): *Cox1*, *Cytb*, *Nd1*, and *Nd3*, encode mitochondrial electron transport chain components (54), supporting persistent impairment of oxidative phosphorylation and mitochondrial function in IVF oocytes. *Bcat2* and *Ethe1* suggest altered mitochondrial nutrient metabolism and the stress-response capacity (55). Importantly, *Aurkc*, a kinase required for meiotic chromosome segregation and oocyte maturation (56), was also downregulated, linking IVF to meiotic dysregulation. Together, these recurrently downregulated genes strengthen the evidence that IVF oocytes exhibit persistent mitochondrial dysfunction and altered meiotic regulation, central mechanisms of oocyte aging and reduced developmental competence.

Because RNA-seq analysis identified pathways related to mitochondrial function, oxidative stress, cytoskeletal organization, and oocyte maturation, we next performed immunofluorescence analyses to validate these signatures at the cellular level. At 12 weeks, oocytes were stained for TOM20 to assess mitochondrial content and organization, α-tubulin to assess spindle structure, and 8-OHdG to assess oxidative DNA damage. IVF oocytes showed reduced mitochondrial occupancy, defined as the percentage of the oocyte area occupied by TOM20^+^ mitochondria, and increased TOM20^+^ particles/μm^2^, reflecting altered mitochondrial distribution or fragmentation. IVF oocytes also showed reduced α-tubulin–defined spindle length, an increase in 8-OHdG^+^ area, and increased whole-oocyte 8-OHdG intensity (Figure 4, I–O). Together, TOM20 staining validated altered mitochondrial content and organization, α-tubulin staining validated cytoskeletal and spindle defects, and 8-OHdG staining validated increased oxidative DNA damage in IVF oocytes, supporting the transcriptomic evidence of mitochondrial dysfunction, altered cellular organization, and oxidative stress.

Taken together, these results demonstrate that IVF is associated with persistent transcriptional and cellular alterations in oocytes, including disrupted mitochondrial metabolism, altered RNA processing, impaired cytoskeletal organization, and increased oxidative stress. These molecular and cellular changes support the conclusion that IVF-conceived female offspring exhibited impaired oocyte quality and accelerated reproductive aging.

### Genes linked to premature reproductive aging are detected in transcriptomes from IVF offspring cumulus cells.

**Figure 5:**
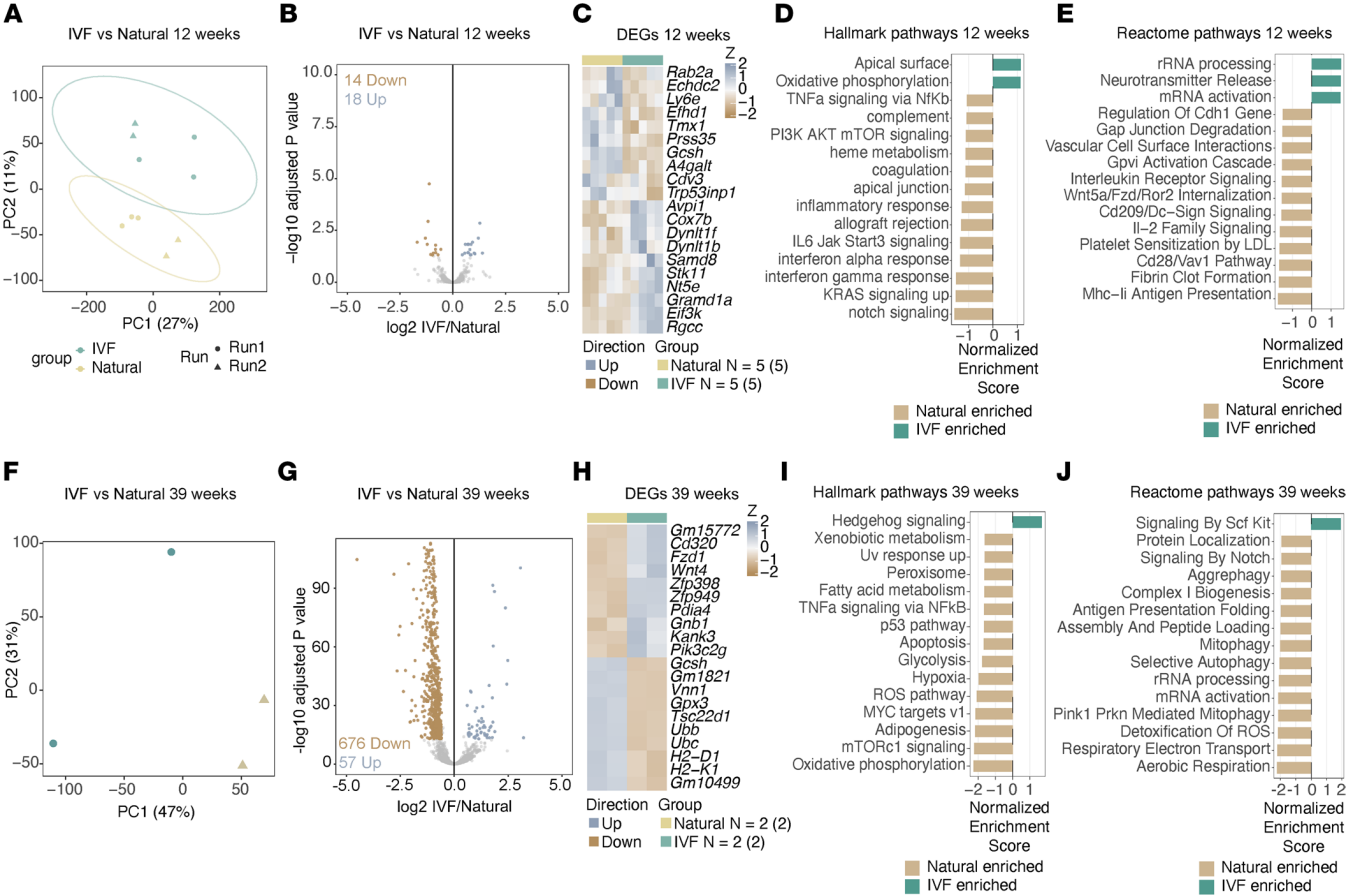
Transcriptome analysis of cumulus cells by RNA-seq at 2 developmental time points. (**A**) PCA of cumulus cell transcriptomes at 12 weeks showing separation between Natural and IVF samples. (**B**) Volcano plot of DEGs in cumulus cells at 12 weeks, with significantly upregulated genes shown in blue and downregulated genes shown in tan. (**C**) Heatmap of the top upregulated and downregulated DEGs at 12 weeks. (**D**) Top enriched Hallmark pathways at 12 weeks. (**E**) Top enriched Reactome pathways at 12 weeks. (**F**) PCA of cumulus cell transcriptomes at 39 weeks showing separation between Natural and IVF groups. (**G**) Volcano plot of DEGs in cumulus cells at 39 weeks. (**H**) Heatmap of the top upregulated and downregulated DEGs at 39 weeks. (**I**) Top enriched Hallmark pathways at 39 weeks. (**J**) Top enriched Reactome pathways at 39 weeks. Heatmaps depict row-scaled normalized expression values, and color bars above heatmaps indicate experimental group assignment. Differential expression analysis was performed using DESeq2, with an adjusted *P* value of less than 0.05. Hallmark and Reactome pathway enrichment analyses were performed using significantly dysregulated genes from each age group.

Cumulus cells, which provide critical support functions for oocyte maturation and reproduction, were analyzed using a SMART-Seq protocol at 12 and 39 weeks. PCA showed partial separation between IVF and Natural cumulus cells at 12 weeks and more limited separation at 39 weeks, consistent with transcriptional heterogeneity between samples (Figure 5, A and F). Differential expression analysis identified 18 upregulated and 14 downregulated genes in IVF cumulus cells at 12 weeks (Figure 5B and Supplemental Table 8), and 57 upregulated and 676 downregulated genes at 39 weeks (Figure 5G and Supplemental Table 9). Heatmaps of the top upregulated and downregulated DEGs are shown at both time points (Figure 5, C and H). These findings suggest that IVF was associated with persistent transcriptional changes in cumulus cells, with a larger number of DEGs detected at 39 weeks.

Pathway enrichment analysis showed that IVF persistently disrupted cumulus cell programs required to maintain the follicular niche. At 12 weeks, the dominant changes pointed to impaired mitochondrial activity, inflammatory activation, and weakened cumulus-oocyte communication (Figure 5, D and E). By 39 weeks, this shifted toward a broader stress phenotype marked by oxidative damage responses, apoptosis, altered metabolism, and impaired mitochondrial quality control (Figure 5, I and J). Together, these findings suggest that IVF reprogrammed cumulus cells toward an aging-like state that may compromise oocyte maturation and follicle maintenance.

Finally, as before, we overlapped our datasets, which revealed 3 upregulated and 8 downregulated DEGs that were common between 12- and 39-week cumulus cells (Supplemental Figure 3, E and F). The upregulated genes included *Nt5e*, which encodes CD73, suggesting altered extracellular adenosine signaling, which is linked to inflammation, tissue remodeling, hypoxia responses, and cellular senescence (57). *Nrcam*, a cell adhesion molecule (58), may reflect altered cell-cell communication within the cumulus-oocyte complex. In contrast, downregulated genes in IVF cumulus cells included *Cdh2*, which suggests altered cell adhesion and impaired cumulus-oocyte communication (59), whereas *Echdc2*, *Aaglat*, and *Tmx1* pointed to disrupted mitochondrial and lipid metabolism (60). Together, these recurrently downregulated genes indicate persistent disruption of cumulus cell adhesion, metabolic support, and stress-response pathways in IVF-conceived female mice.

Together, these results indicate that IVF is associated with altered cumulus cell transcriptional programs involving inflammation, cell junction regulation, mitochondrial metabolism, oxidative stress, cell signaling, and antigen presentation. Because cumulus cells regulate oocyte metabolism and oocyte–somatic cell communication, these changes may contribute to impaired follicular support and reduced oocyte quality in IVF-conceived females.

### IVF offspring exhibit altered ovarian DNA methylation.

**Figure 6:**
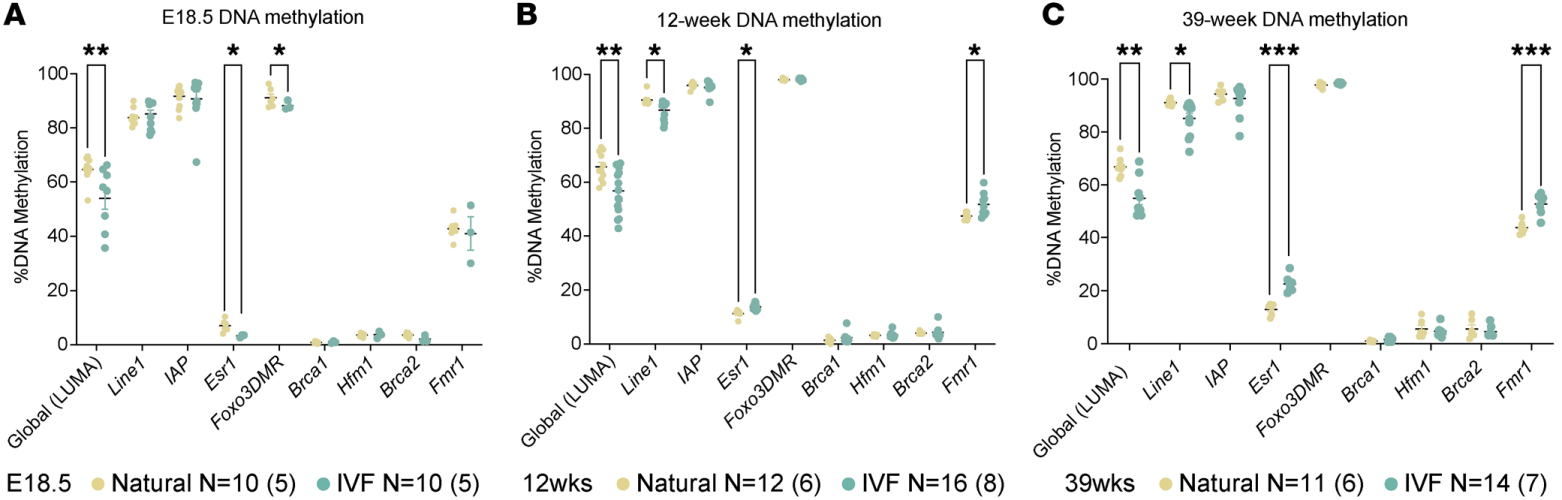
DNA methylation analysis of ovaries at 3 developmental time points. Global DNA methylation was assessed using LUMA, and locus-specific DNA methylation was analyzed by targeted bisulfite sequencing in whole ovaries (see Methods for CpG coverage and primer information). DNA methylation was evaluated at repetitive elements (LINE1 and IAP), promoter regions of *Esr1*, *Brca1*, *Hfm1*, *Brca2*, and *Fmr1*, and at the intergenic differentially methylated region (DMR) of *Foxo3*. (**A**) DNA methylation analysis at E18.5. (**B**) DNA methylation analysis at 12 weeks. (**C**) DNA methylation analysis at 39 weeks. Each dot represents 1 biological replicate. Sample sizes are indicated below each panel as the number of animals (litters). Data are presented as the mean ± SEM. Statistical significance between Natural and IVF groups was determined using an unpaired, 2-tailed *t* test. *P < 0.05, **P < 0.01, and ***P < 0.001 compared with Naturals.

Because IVF is associated with changes in DNA methylation in multiple tissues (5, 22) and reproductive aging can be assessed through changes in DNA methylation, we interrogated global DNA methylation using a luminometric methylation assay (LUMA) on ovary DNA at E18.5, 12 weeks, and 39 weeks. We observed statistically significant decreased DNA methylation in ovaries from IVF-conceived female offspring compared with Naturals at all time points (Figure 6, A–C). Additionally, loss of DNA methylation at transposable elements (TEs) has previously been linked to oocyte aging and follicle depletion (61). Accordingly, we measured DNA methylation changes in LINE1 and Intracisternal A-type Particle (IAP) elements, 2 major TEs in the mouse genome, using a targeted bisulfite sequencing assay. We observed a decrease in LINE1 DNA methylation in both 12- and 39-week-old IVF offspring compared with Naturals (Figure 6, A–C), with no changes detected in IAP elements (Figure 6, A–C).

To determine whether gene expression changes were associated with altered DNA methylation, we performed targeted bisulfite sequencing at the promoters of genes linked to POI and differentially expressed across ages and sample types (*Esr1*, *Foxo3*, *Brca1*, *Hfm1*, *Brca2*, and *Fmr1*). DNA methylation at *Esr1* was significantly decreased at E18.5 (Figure 6A), which may contribute to increased germ cell death during niche breakdown, and was significantly increased at 12 and 39 weeks (Figure 6, B and C), correlating with downregulated expression and decreased serum estradiol levels. *Fmr1* methylation was also elevated at 12 and 39 weeks (Figure 6, B and C). Hypermethylation of *Esr1* and *Fmr1* is associated with menopause and senescence (62, 63), consistent with our phenotype. Because ovarian heterogeneity may mask cell type–specific transcription and DNA methylation changes, there may be an underestimation of observed differences. Taken together, these results suggest that DNA methylation dysregulation is a candidate driver for the observed ovarian phenotypes, consistent with our previous findings in other tissues in which altered DNA methylation mediates IVF-associated adverse outcomes (11, 24).

### IVF affects the number of pups and resorptions in pregnancies from IVF-conceived female offspring.

**Figure 7:**
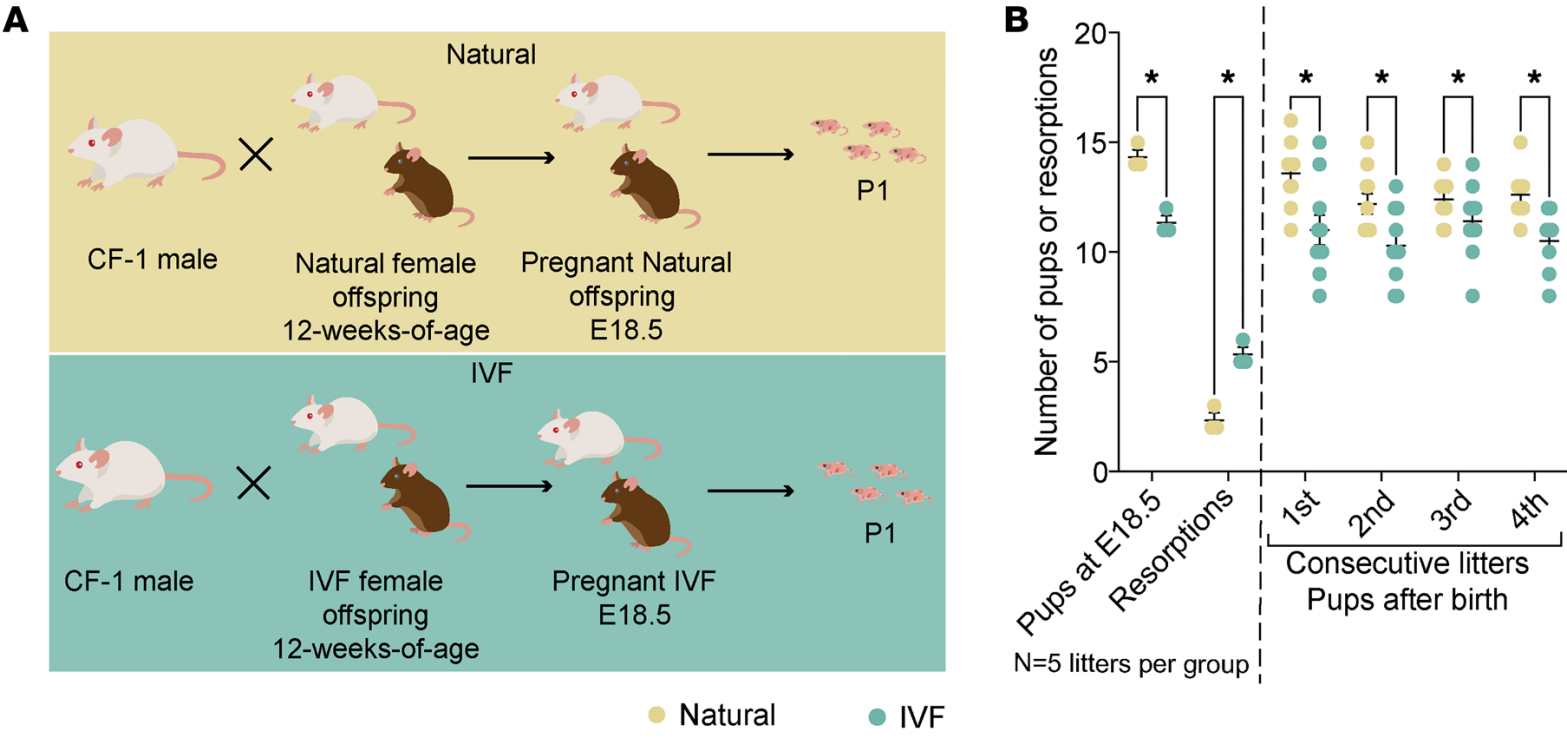
Number of pups and resorption rates for IVF-conceived female offspring. (**A**) Experimental design and mouse model. Top: F2 Naturally conceived offspring were generated by mating CF-1 male mice with naturally conceived CF-1 female offspring at 12 weeks of age. Bottom: F2 IVF-conceived offspring were generated by mating CF-1 male mice with IVF-derived female offspring at 12 weeks of age. The different coat colors shown in the schematic represent the mixed genetic background of the offspring. Pregnant females from both groups were analyzed at E18.5, and concepti were collected following c-section. Additional breeding pairs were allowed to deliver naturally, and litter size was recorded at P1. (**B**) Number of pups at E18.5, number of resorptions, and total pups born across 4 consecutive litters. Each dot represents 1 independent litter from a separate breeding pair. Data are presented as the mean ± SEM. Sample sizes: *n* = 5 breeding pairs/litters per group for E18.5 and resorption analyses; *n* = 4 consecutive litters per breeding pair for postnatal litter analyses. Statistical significance between natural and IVF groups was determined using an unpaired, 2-tailed *t* test. *P < 0.05, compared with Naturals.

Because gene expression and ovarian health were disrupted in IVF-conceived female mice, we examined the fertility and pup ratio of matings with IVF-conceived female offspring. Twelve-week-old IVF- and naturally conceived female mice were mated with CF-1 WT male mice, and concepti were either collected by c-section at E18.5 (Figure 7A) or allowed to naturally drop. Time to conception and gestation length (19 days) were comparable between the groups. In contrast, pregnancies in IVF-conceived females showed a higher number of resorptions (Figure 7B), fewer live pups 12 hours after birth (Figure 7B), and reduced litter sizes across 4 consecutive litters (Figure 7B). These results indicate that the changes observed in IVF female offspring had an effect on the number of pups and resorptions that was probably due to decreased oocyte quality.

## Discussion

In this study, we use a mouse model to demonstrate that IVF perturbs ovarian development and accelerates reproductive aging in female offspring, with changes in the number of pups and resorptions. These findings extend prior observations of ART-induced alterations in placental development, metabolism, and male reproductive health (4, 6, 24, 64) and provide new insight into how IVF affects the female germline and associated cells throughout life.

**Figure 8:**
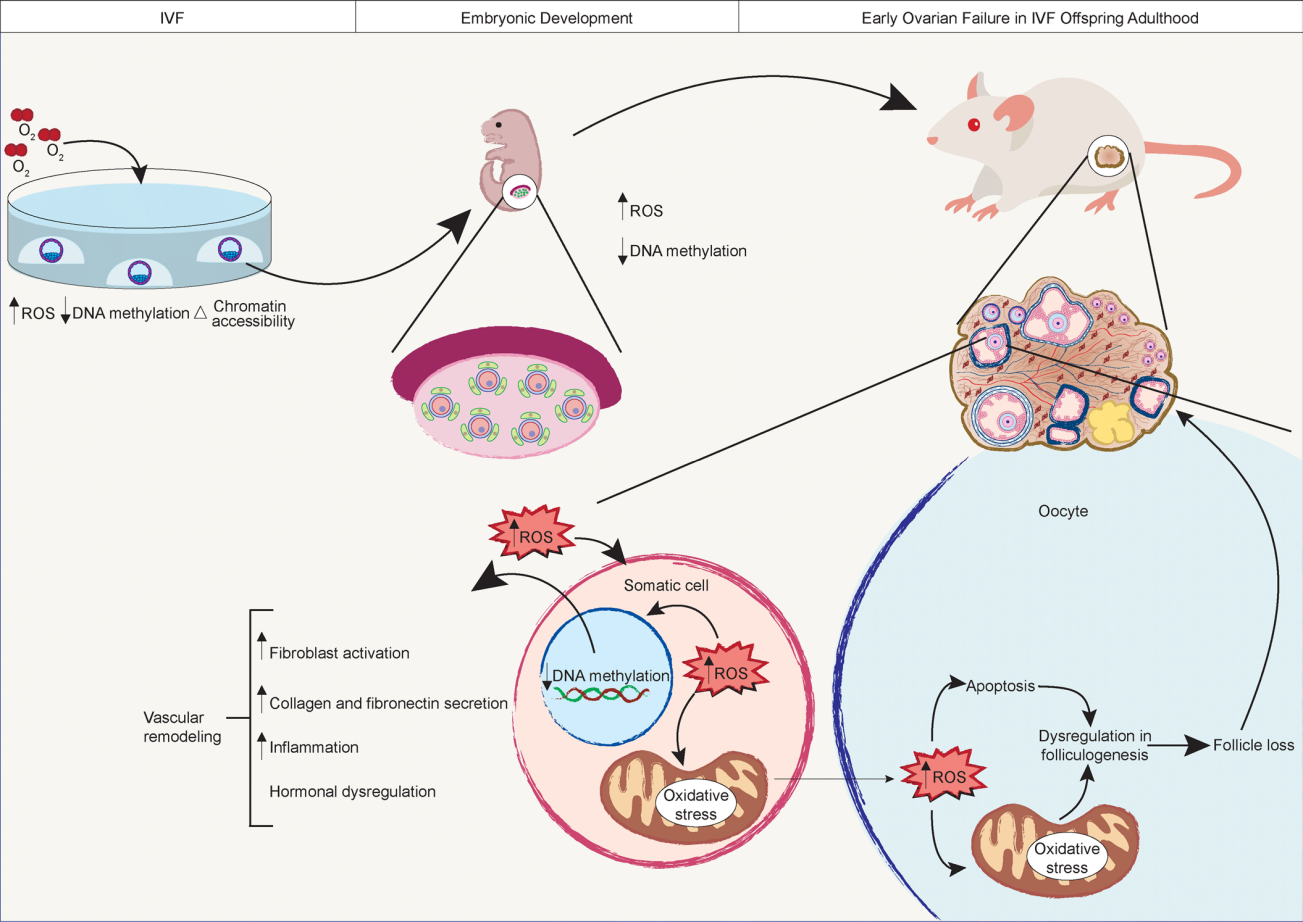
Proposed model of IVF-induced ovarian aging. IVF embryo culture induces early oxidative and epigenetic stress, including increased ROS, altered DNA methylation, and chromatin changes. These perturbations persist across development and contribute to ovarian dysfunction in adult offspring, including somatic cell stress, vascular remodeling, inflammation, altered follicle regulation, oocyte oxidative stress, apoptosis, and follicle loss.

A central theme emerging from our data is that IVF acts as a prenatal environmental exposure that reprograms ovarian biology (Figure 8). In humans, IVF-conceived female offspring appear to be at higher risk for bone aging abnormalities and exhibit changes in luteal hormone levels (14). Here, we show that the hallmarks of premature reproductive aging, including reduced follicle counts, disrupted hormone production, and POI-associated gene expression, were evident in IVF-conceived females. Similar features have been observed in human POI, as well as in models of maternal obesity and inflammation, supporting the idea that IVF mimics other adverse intrauterine environments in depleting the ovarian reserve (65). Importantly, we found that IVF-induced changes converged on pathways regulating metabolism, cell division, and folliculogenesis, raising the possibility that suboptimal conditions during early embryogenesis initiate a cascade that compromises ovarian function throughout life.

Our temporal clustering analyses revealed that IVF fundamentally altered age-dependent gene expression trajectories in ovaries and oocytes compared with naturally conceived females. While the Natural groups showed predictable developmental transitions, such as declining expression of cell-cycle and chromosomal pathways with age and stable expression of core metabolic regulators, IVF-conceived offspring exhibited significant deviations from these trajectories. Clusters that normally remained stable became dysregulated, with premature upregulation of DNA replication and cell-cycle pathways in cumulus cells and persistent activation of metabolic programs in both oocytes and somatic compartments. Conversely, clusters that typically increased with age, such as those supporting germ cell development and fertilization, were reduced in IVF, suggesting an early depletion of follicular support mechanisms. Together, these shifts reflect a reprogramming of ovarian developmental timing, with premature activation of stress and metabolic responses coupled to early loss of pathways required for long-term oocyte quality. Such remodeling of temporal expression dynamics provides a mechanistic framework for how IVF accelerates reproductive aging. Importantly, IVF oocytes showed reduced TOM20 mitochondrial occupancy, increased cytoplasmic particle density, shorter spindle length, and increased 8-OHdG signal. These findings indicate that the transcriptional signatures of mitochondrial dysfunction, cytoskeletal disruption, and oxidative stress were reflected at the cellular level, providing direct evidence that IVF compromises oocyte quality.

IVF also appeared to affect cumulus cells that support oocyte maturation. Cumulus cells coordinate nutrient transfer, oocyte energetic support, cell-cell communication, and meiotic competence. In IVF-derived cumulus cells, the dominant changes pointed to inflammatory activation, impaired metabolic support, disrupted junctional communication, and reduced mitochondrial quality control, suggesting persistent dysfunction of the follicular microenvironment. These changes provide a mechanistic link between IVF-associated somatic cell dysfunction and the reduced follicle reserve, altered hormone levels, impaired oocyte quality, and accelerated ovarian aging observed in IVF-conceived females.

Our results also suggest that epigenetic dysregulation is one major mechanism linking IVF to premature reproductive aging. Altered DNA methylation at *Esr1* and *Fmr1*, together with global hypomethylation and dysregulation of LINE1, are signatures of accelerated epigenetic aging (66). Additionally, hypomethylation of LINE1 elements is linked to its transcriptional activation, accumulation of DNA damage, and oocyte loss through apoptosis (35). In humans, similar patterns are associated with ovarian insufficiency, menopause, and infertility (35). These changes are consistent with the increased LINE1 and ORF1p protein levels we observed by Western blotting. However, because LINE1 DNA methylation and ORF1p protein levels were measured in whole ovaries, we could not determine whether increased LINE1 protein expression originated from germ cells, follicular somatic cells, or other ovarian cells. Although LINE1 expression showed a nonsignificant trend toward increased expression in isolated oocytes and cumulus cells, future cell type–specific analyses will be required to define the cellular source of LINE1 activation in IVF ovaries.

We observed changes in both the morphology and molecular status of the entire ovary and our analyses of oocytes and cumulus cells underscore the vulnerability of both germ and somatic ovarian compartments to IVF. Disruption of spindle formation, energy metabolism, and cumulus-oocyte communication pathways is similar to findings from obesity, alcohol, and diabetes exposure models, all of which compromise fertility (67–69).

Together, the data presented here support a model in which IVF-induced stress during early embryogenesis reprograms both germ cell and somatic ovarian compartments. The absence of differences in germ cell numbers at E18.5, followed by postnatal follicle loss, suggests that IVF does not reduce the initial fetal germ cell pool but instead accelerates postnatal ovarian decline. This decline is associated with impaired mitochondrial function, oxidative stress, spindle abnormalities, altered cumulus-oocyte communication, endocrine disruption, vascular impairment, apoptosis, and epigenetic instability. Many of these pathways are also central features of biological aging (70), suggesting that IVF accelerates systemic aging trajectories.

Despite the increasing use of ART in humans, long-term studies of reproductive and metabolic health remain limited, especially in women. A limitation of this study is the absence of direct validation in human ART-derived samples. However, mouse IVF models have been highly informative for translational studies, as they have anticipated several adverse outcomes later observed in humans, including altered fetal and placental growth, metabolic dysfunction, cardiovascular changes, and imprinting-related abnormalities. Important differences still exist between mouse IVF conditions and clinical ART protocols, including species-specific differences in ovarian stimulation, embryo culture media, the duration of culture, and the use of vitrification. Therefore, our findings should not be interpreted as directly equivalent to current clinical IVF outcomes, but rather as mechanistic evidence from a controlled model system with strong translational relevance that establishes a foundation for future human studies.

Future studies using discarded immature oocytes or cumulus cells from patients who have undergone IVF will be important to determine whether the transcriptomic, epigenetic, and cellular signatures identified here are conserved in humans. Defining how IVF conditions influence oocyte epigenetic integrity, mitochondrial function, oxidative stress, and ovarian somatic cell health may help guide strategies to mitigate premature reproductive aging and improve fertility outcomes in ART populations. Ultimately, our findings underscore the importance of monitoring not only immediate pregnancy outcomes but also the lifelong reproductive health trajectories of ART-conceived individuals.

## Methods

### Sex as a biological variable.

For this study, sex was considered a biological variable in the experimental design because of the sexually dimorphic adverse outcomes previously reported (4, 16). We focused exclusively on female mice to enable a comprehensive evaluation of the effects of IVF on the onset of ovarian insufficiency and premature reproductive aging, while prior work from our laboratory has characterized the effect of IVF on the male reproductive system (24). These results should not be generalized across sexes, as reproductive development differs between males and females mammals, and the adverse outcomes associated with IVF appear to be sex specific.

### Animals.

Breeding stocks of CF-1 females and CD-1 vasectomized males (Charles River Laboratories) and B6/SJL males (The Jackson Laboratory) were maintained under pathogen-free conditions in polysulfone cages on a 12-hour light/12-hour dark cycle, with ad libitum access to water and chow (Laboratory Autoclavable Rodent Diet 5010, LabDiet).

### Generation of natural offspring.

Naturally conceived offspring (natural) were obtained by mating 8-week-old CF-1 females, during their natural estrous cycle, with 8-week-old B6/SJL males. The presence of a vaginal plug was recorded as E0.5, and embryos were allowed to develop entirely in vivo without embryo transfer (Figure 1A).

### Generation of IVF offspring.

As previously described (24), IVF offspring were generated according to optimized protocols recommended by The Jackson Laboratory (71). Eight-week-old CF-1 female mice were superovulated with 5 IU equine Chorionic Gonadotropin (eCG), followed 46 hours later by 5 IU human chorionic gonadotropin (hCG). On the day of IVF, sperm were collected from the vas deferens and cauda epididymis of B6/SJL males into EmbryoMax Human Tubal Fluid (HTF) medium (EMD Millipore) supplemented with 3% w/v BSA (AlbuMax, Gibco, Thermo Fisher Scientific) and maintained under mineral oil (Irvine Scientific). Sperm were capacitated for at least 1 hour prior to fertilization. Oocytes were harvested and inseminated with capacitated sperm in HTF medium. After 4 hours, fertilized oocytes with visible pronuclei were washed through HTF and then into EmbryoMax KSOM medium supplemented with half-strength amino acids (KSOM+AA, EMD Millipore), before being cultured to the blastocyst stage under mineral oil at 37°C in 5% CO_2_, 5% O_2_, and 90% N_2_. After 3.5 days of culture, blastocysts were washed in Multipurpose Handling Medium Complete (MHM-C) (Irvine Scientific) containing gentamicin prior to embryo transfer. Pseudopregnant recipients (postcopulation day 3.5) were generated by mating CF-1 females with CD-1 vasectomized males, and each recipient was given10 blastocysts by nonsurgical embryo transfer (Figure 1B). The day of blastocyst transfer was designated as E3.5.

### Cesarean delivery and fostering.

At E18.5 for natural pregnancies and E19.5 for IVF pregnancies, pregnant dams were euthanized, and cesarean sections (c-sections) were performed to collect fetuses and placentas. The adult cohorts analyzed in the present study were generated from pups delivered by c-section in the previously described cohorts (4, 16). These pups were fostered to WT CF-1 dams, and litters were standardized to 10 pups per dam until weaning to control for the postnatal nutritional environment. After weaning, the females were housed at 5 animals per cage for both the 12- and 39-week cohorts. Birth weight and early postnatal growth parameters for these cohorts were previously reported in those studies.

### Tissue collection.

At E18.5 and at 12 or 39 weeks of age, naturally mated females or pseudopregnant recipient mice carrying IVF embryos were euthanized, and 1 ovary was snap-frozen in liquid nitrogen, while the other was fixed in 10% phosphate-buffered formalin for histological analysis. For adult cohorts, females used for serum hormone measurements were euthanized at proestrus to control for estrous cycle–dependent variation in endocrine parameters. Blood was collected, and serum was isolated by centrifugation at 4°C and stored at –80°C for hormone measurements.

### Whole embryonic ovary staining.

Fixed ovaries at E18.5 were stained for germ cell counting using a previously described protocol (31). Briefly, after 24 hours of fixation, gonads were washed in fresh PBS for 30 minutes at room temperature on a laboratory shaker (100 rpm). Ovaries were then incubated in blocking solution (2% BSA, 0.5% Triton X-100, and 15% goat serum in PBS) for 3 hours at room temperature with shaking. Following blocking, ovaries were incubated for 4 days with anti-GCNA1 (1:100, ab82527, Abcam) diluted in blocking solution, under the same conditions. After primary antibody incubation, ovaries were washed 4 times for 15 minutes in blocking solution without goat serum to remove excess antibody. Samples were then incubated for 2 days with a donkey anti–rat IgG (H+L) highly cross-adsorbed secondary antibody conjugated to Alexa Fluor 555 (1:1,000, catalog A78945, Thermo Fisher Scientific) in blocking solution. Ovaries were washed 3 times for 30 minutes in blocking solution without goat serum, followed by incubation with DAPI (1:100, Thermo Fisher Scientific) for 30 minutes in blocking solution. After nuclear staining, ovaries were washed in PBS and cleared in Scale CUBIC-1 solution (solution prepared in-house) for 24 hours at room temperature. CUBIC-1 is an aqueous tissue-clearing reagent that reduces light scattering and improves optical penetration, allowing 3D imaging of intact ovaries. Scale CUBIC-1 solution was prepared using 125 g urea (CAS 57 13 6), 156 g Quadrol 80% (CAS 102 60 3), and 144 g water, followed by addition of 75 g Triton X-100 (CAS 9036 19 5) after dissolution. Imaging was performed on a Leica SP8 confocal microscope, acquiring 3D scans of whole ovaries with a *z*-step size of 5 μm over 30 minutes. Germ cell quantification from 3D ovarian visualizations was performed using Imaris 9.7 software (Bitplane). All analyses were conducted in a blinded manner, with samples coded and evaluated by an independent reviewer to prevent bias.

### H&E and PR staining.

Ovarian sections were stained with H&E as previously described (72). Stained sections were imaged using an EVOS FL Auto Cell Imaging System and software (Thermo Fisher Scientific) at ×4 magnification. Follicular counts were performed as previously described (72) by 2 blinded individuals. To avoid repeated counting of the same follicle, every tenth serial section throughout the entire ovary was analyzed, and only follicles containing a clearly visible oocyte nucleus were counted. Follicles were classified by developmental stage as previously described (72), and counts from all analyzed sections were summed and expressed as the total number of follicles per ovary.

To assess changes in collagen deposition and potential fibrosis, PR staining was used on ovarian sections as previously described (22). Briefly, tissue sections were deparaffinized in xylene and rehydrated through a graded series of ethanol and then immersed in a PR staining solution (Picrosirius Red F3B Direct Red 80, C.I. 35782, MilliporeSigma, in a 1.3% saturated aqueous picric acid solution, MilliporeSigma,). After a 30-minute incubation in the staining solution at room temperature, the slides were de-stained with 0.05 N HCl. The tissue sections were then dehydrated in 100% ethanol, cleared in xylene, and mounted with Permount Mounting Medium (Thermo Fisher Scientific). Stained slides were imaged using an EVOS FL Auto Cell Imaging System (Thermo Fisher Scientific). The percentage of PR^+^ signal was calculated with ImageJ (NIH) using 3 different testicular sections per animal.

### Ovarian IHC.

For ovarian IHC analysis, ovaries were treated as previously published (24). Briefly, histological sections were rehydrated using xylene, followed by graded series of ethanol and then with PBS. Antigen retrieval was performed using Antigen Unmasking Solution (Vector Laboratories). Sections were subsequently washed, quenched with a 30% hydrogen peroxide/methanol solution, washed again in PBS, and blocked using 15% normal goat serum in 0.3% Triton-PBS for 1 hour at room temperature. The primary antibodies anti-CD31 (ab182981, Abcam) and anti–cleaved caspase 3 (catalog 9664, Cell Signaling Technology) were applied at a dilution of 1:100 for 1 hour at room temperature, followed by overnight incubation at 4°C. Negative control slides received 15% normal goat serum without primary antibodies. The following day, slides were washed with PBS and secondary antibodies, goat anti–rabbit IgG (H+L) Highly Cross-Adsorbed Secondary Antibody and Alexa Fluor Plus 647 (catalog A32728, Thermo Fisher Scientific), were applied at 1:250 for 1 hour. After washing with PBS, slides were incubated with DAPI (Thermo Fisher Scientific) at 1:100 in blocking solution for 30 minutes. Finally, slides were washed in PBS and distilled water, and then mounted using ProLong Diamond Antifade Mountant (Thermo Fisher Scientific).

### Oocyte staining and immunofluorescence.

For immunostaining, denuded oocytes were fixed in 4% paraformaldehyde in Dulbecco’s PBS (DPBS) containing 0.1% Triton X-100 for 30 minutes at room temperature, permeabilized in DPBS containing 0.5% Triton X-100 for 30 minutes, and incubated in blocking solution (15% goat serum in DPBS containing 0.1% Triton X-100) for 1 hour. Oocytes were then incubated overnight at 4°C with primary antibodies diluted in blocking solution using one of the following combinations: TOM20 (1:200, ab186735, Abcam) and α-tubulin (1:100, catalog MA1-19401, Thermo Fisher Scientific), or 8-OHdG (1:100, catalog BS-1278R, Thermo Fisher Scientific) and α-tubulin (1:100, Thermo Fisher Scientific). Oocytes were rinsed in DPBS and incubated with Alexa Fluor 488– or 647–conjugated secondary antibodies (1:1,000, Invitrogen, Thermo Fisher Scientific) in blocking solution for 2 hours. Nuclei were counterstained with DAPI (MilliporeSigma). Stained oocytes were imaged using a Leica SP8 laser scanning confocal microscope with a ×40 water apochromat objective. 3D confocal *Z*-stacks were acquired for each oocyte. 3D visualization and spindle length measurements were performed using Imaris 9.7 software (Bitplane AG). Only spindles that were completely visible and oriented in an appropriate plane for accurate measurement were included. TOM20 and 8-OHdG fluorescence intensities were quantified using Fiji/ImageJ with antibody-specific thresholds that were kept constant across Natural and IVF groups. All measurements were performed in a blinded manner.

### Estradiol and progesterone levels.

Whole blood was collected by cardiac puncture on the day of euthanasia. Samples were centrifuged and serum was collected and stored a –80°C. Estradiol was measured using a Mouse Estradiol ELISA kit (Abcam), and progesterone was measured using a Mouse Progesterone ELISA kit (Crystal Chem) following the manufacturers’ instructions.

### DNA and RNA isolation from ovaries.

DNA and RNA were isolated from one-half of each snap-frozen ovary or a whole E18.5 gonad, as previously described (4). Briefly, for DNA, tissues were digested in lysis buffer (50 mM Tris, pH 8.0, 100 mM EDTA, 0.5% SDS) with proteinase K (180 U/mL; MilliporeSigma) overnight at 55°C. Genomic DNA was isolated using phenol/chloroform/alcohol (25:24:1) (MilliporeSigma), followed by ethanol precipitation and resuspension in TE buffer (10 mM Tris-HCl, pH 8.0, 0.5 mM EDTA).

RNA was isolated using an adapted protocol from the TRIzol (Thermo Fisher Scientific) and Qiagen Micro RNA Kit (Qiagen) following the manufacturer’s protocol. DNAse treatment was performed during RNA isolation to eliminate genomic DNA contamination. RNA quality and concentration were determined by RNA ScreenTape analysis using a TapeStation (Agilent Technologies).

### Luminometric methylation assay.

Genomic DNA (1 μg) from ovaries was used to measure global DNA methylation by luminometric methylation assay as previously described (73).

### Locus-specific DNA methylation analysis using targeted next-generation bisulfite sequencing.

DNA methylation levels at *Esr1*, *Foxo3*, *Brca1*, *Hfm1*, *Brca2*, *Fmr1* LINE1 and IAP genomic regions were assessed using targeted DNA methylation analysis via next-generation sequencing. Primer sequences are provided in Supplemental Table 10. The assay was performed as previously described (74).

### Immunoblotting.

Half of the ovaries from 12- and 39-week females were used for protein analysis by Western blotting as previously described (4, 24). Briefly, ovaries were lysed in RIPA buffer (Cell Signaling Technology) containing protease inhibitors without EDTA (Merck Millipore). Protein lysates (20 μg/sample) were separated and probed with primary antibodies diluted in 5% nonfat dry milk in TBST: anti-tubulin (1:1,000; catalog 3873, Cell Signaling Technology), anti-CD31 (1:100; Abcam), anti–cleaved caspase 3 (1:200; Cell Signaling Technology), anti–ORF1p-LINE1 (1:500; ab216324, Abcam), and anti-TOM20 (1:1,000; Abcam). Levels of CD31, cleaved caspase 3, ORF1/p-LINE1, and TOM20 were quantified relative to GAPDH (1:10,000; Cell Signaling Technology) and compared between groups. For quantitative analysis, target protein intensities were first normalized to GAPDH within each lane. To account for inter-blot variability across independent experiments, normalized values from each blot were subsequently scaled to the mean intensity of the Natural group within the corresponding blot prior to pooling for statistical analyses.

### mtDNA copy number.

Ovary mtDNA copy numbers were estimated using RT-qPCR as previously described (75). Briefly, previously extracted DNA was amplified using primers targeting 2 mitochondrially encoded genes, *ND1* and *Cox3* (Supplemental Table 11), and the nuclear encoded small subunit 18S rRNA (18S). RT-qPCR reactions were performed using the QuantStudio 7 Flex Real Time PCR System (Life Technologies, Thermo Fisher Scientific) with Power SYBR Green Master Mix (Applied Biosystems), using the optimized cycling parameters detailed in Supplemental Table 11. Relative mtDNA copy numbers were determined by calculating the difference in Ct values between the mitochondrial targets (*ND1* and *Cox3*) and the nuclear reference gene (18S) for each sample (ΔCt). The average ΔCt values of the mitochondrial genes were then normalized to the mean Natural control group using the ΔΔCt method, and data are presented as relative mtDNA/nDNA ratios.

### RNA-seq.

We performed RNA-seq on a random subset of E18.5, 12- and 39-week ovaries from 5–6 individuals for each group and different litters to determine changes in gene expression. As previously described (4, 16, 22), total RNA (4 μg) was used to prepare mRNA-seq libraries using the KAPA mRNA-Seq library synthesis kit and the KAPA Single-Indexed adapter kit (Kapa Biosystems). Library quality control was conducted using High Sensitivity DNA ScreenTape for TapeStation (Agilent Technologies) and Kapa Library Quantification Kit (Kapa Biosystems). Sequencing was performed using the NovaSeq 1000 platform (Illumina).

RNA-seq reads were analyzed as previously described (4). The raw sequencing data reported in this work have been deposited in the NCBI’s Gene Expression Omnibus (GEO) database (GEO GSE307881). Count data were analyzed in R (version 4.4.1) using a combination of Bioconductor and CRAN packages. Differential expression analysis was performed with DESeq2, whereas temporal clustering of gene expression trajectories was assessed using a WCSS approach, as previously described (34).

Briefly, to identify coordinated temporal transcriptional programs across ovarian development and aging, we applied an unsupervised clustering approach based on WCSS analysis. Raw count matrices were first subjected to stringent expression filtering, in which genes were retained only if they reached a cpm of 1 or greater (computed with edgeR:cpm) in at least 20% of all samples (minimum of 2 samples) and were detected in at least 1 sample within each age group. Counts passing these filters were converted to cpm values and transformed into gene-wise *z* scores by mean centering and variance scaling each gene. For each gene, average expression values were calculated for each developmental stage to generate a gene by age expression profile matrix used for temporal clustering.

*K*-means clustering was then performed to group genes with similar temporal expression trajectories across E13.5 PGCs, 12-week oocytes, and 39-week oocytes. To determine the optimal number of temporal clusters, *k*-means clustering (*Rk*-means, nstart = 50, iter.max = 1,000, fixed seed) was performed across *k* = 2–15, and the WCSS was calculated for each *k* value. WCSS quantifies the variability of genes within each cluster relative to the cluster centroid, where lower WCSS values indicate tighter and more homogeneous clustering. The optimal number of clusters was selected using the elbow criterion, defined as the point beyond which increasing the number of clusters resulted in only marginal reductions in WCSS. Relative improvements between successive *k* values were also evaluated, favoring the first *k* with less than a 5% additional reduction in WCSS. Natural samples were used as the training group to define baseline temporal transcriptional trajectories, and IVF samples were subsequently projected onto these clusters to evaluate alterations in developmental gene expression dynamics associated with IVF. Functional enrichment of clustered and DEGs was assessed using GO overrepresentation analysis. Changes in temporal cluster membership between groups were visualized using alluvial plots, and overlaps across datasets were examined using the VennDiagram package.

### Low-input RNA-seq for single oocytes and cumulus cells.

We performed RNA-seq on a subset of PGCs, single oocytes and cumulus cells from E13.5, 12-week-old, and 39-week-old mice. PGCs were collected as previously described (74). For oocytes, 72 hours before their 12- or 39-week birth date, IVF- and naturally conceived female offspring were superovulated with 5 IU eCG, followed by 5 IU hCG 46 hours later. The next day, cumulus-oocyte complexes (COCs) were collected from the superovulated animals and deposited in MHM-C under mineral oil containing 10 μg/mL hyaluronidase (MilliporeSigma) and incubated at 37°C in an atmosphere of 5% CO_2_, 5% O_2_, and 90% N_2_ for 2 minutes. Then, the cleaned oocytes were washed 3 times in fresh MHM-C drops and individually snap-frozen in 2 μL target cell lysis (TCL) lysis buffer (2× TCL, Qiagen) with 1% 2-mercaptoethanol (Bio-Rad) in low-binding PCR tubes. A similar procedure was followed for cumulus cells, except they were counted using a hemocytometer to ensure 100 cells per 2 μL solution before freezing. RNA-seq libraries were constructed following a modified SMART-Seq protocol (47). Briefly, after cell lysis, RNA was isolated using RNAClean-XP beads (Beckman Coulter), and full-length polyadenylated RNA was reverse-transcribed using Superscript II (Invitrogen, Thermo Fisher Scientific). The cDNA was amplified using 10 cycles and subsequently used (0.33 ng) to construct a pool of uniquely indexed samples with the Nextera XT kit (Illumina). Library quality control was conducted using High Sensitivity DNA ScreenTape for TapeStation (Agilent Technologies) and a Library Quantification Kit (KAPA Biosystems). Finally, pooled libraries were sequenced on a NextSeq 1000. Data were analyzed as shown before (47). Briefly, raw reads were trimmed with trimgalore and aligned to the mm10 reference *Mus musculus* genome using Star. A count table was generated using featureCounts and used as input for the R package DESeq2. Finally, pathway analyses were performed using the clusterProfiler R package.

### Statistics.

For all samples, normality was assessed using the Shapiro-Wilk test. Data that did not meet the normality assumption were analyzed using the nonparametric Mann-Whitney *U* test, whereas normally distributed data were analyzed using an unpaired 2-tailed *t* test with Welch’s correction. Unless otherwise indicated, each data point represents 1 individual animal or biological sample. For adult cohorts, no more than 2 littermates per litter were included, and these animals were randomly selected to minimize litter effects. The number of animals and independent litters analyzed is indicated in each figure. Western blot analyses were performed using an unpaired, 2-tailed Welch’s *t* test following normalization to account for unequal variance between groups. Probability of genomic regions in the array were compared with a Bernoulli distribution using R version 4.2.2 (R Foundation for Statistical Computing; www.R-project.org/). Significant differences comparing Natural and IVF offspring were considered statistically significant when the *P* value was less than 0.05. Differences in variability were calculated using *F* tests, with statistically significant differences comparing the natural and IVF groups denoted when the *P* value was less than 0.05. All statistical analyses were performed using GraphPad Prism 11 (GraphPad Software). All data are presented as mean ± SEM.

### Study approval.

All animal work was conducted with the approval of the IACUC at the University of Pennsylvania. IACUC protocol 80354 has been previously revised and approved.

### Data availability.

All values for all data points in graphs are reported in the Supporting Data Values file. Additionally, the data array and sequencing data underlying this article are available in the GEO database (GEO GSE307881).

## Author contributions

EARC and MSB designed research studies. EARC, CNH, AJS, ADM, CJK, ZL, and LR conducted experiments. EARC, AJS, ADM, and CJK acquired data. NP provided the confocal microscopy equipment and resources necessary for imaging. RMS participated in project development and provided critical feedback. EARC wrote the manuscript, and all authors edited and reviewed the manuscript prior to submission. EARC is listed as first author on the basis of his conceptualization and initiation of the project.

## Conflict of interest

The authors have declared that no conflict of interest exists.

## Funding support

This work is the result of NIH funding, in whole or in part, and is subject to the NIH Public Access Policy. Through acceptance of this federal funding, the NIH has been given a right to make the work publicly available in PubMed Central.

- National Center for Translational Research in Reproduction and Infertility grants P50 HD068157 (to MSB); R01 HD102013 (to NP); F32 HD107914 (to ERC); and F31 HD117524 (to CNH).
- The National Institute of General Medical Sciences grant R01 GM139970 (to NP).

## Supplementary Material

Supplemental data

Unedited blot and gel images

Supplemental table 1

Supplemental table 10

Supplemental table 11

Supplemental table 2

Supplemental table 3

Supplemental table 4

Supplemental table 5

Supplemental table 6

Supplemental table 7

Supplemental table 8

Supplemental table 9

Supporting data values

## Version 1 06/30/2026

In-Press Preview

## Version 2 08/17/2026

Electronic publication
