# Supplementary material for: Mouse offspring conceived by in vitro fertilization exhibit accelerated reproductive aging through premature ovarian insufficiency
Source: J Clin Invest. 2026 Jun 30;136(16):e201633. doi: 10.1172/JCI201633 (PMC13476136; doi:10.1172/JCI201633)
Supplement: Supplementary file 1 [file jci-136-201633-s142.pdf]

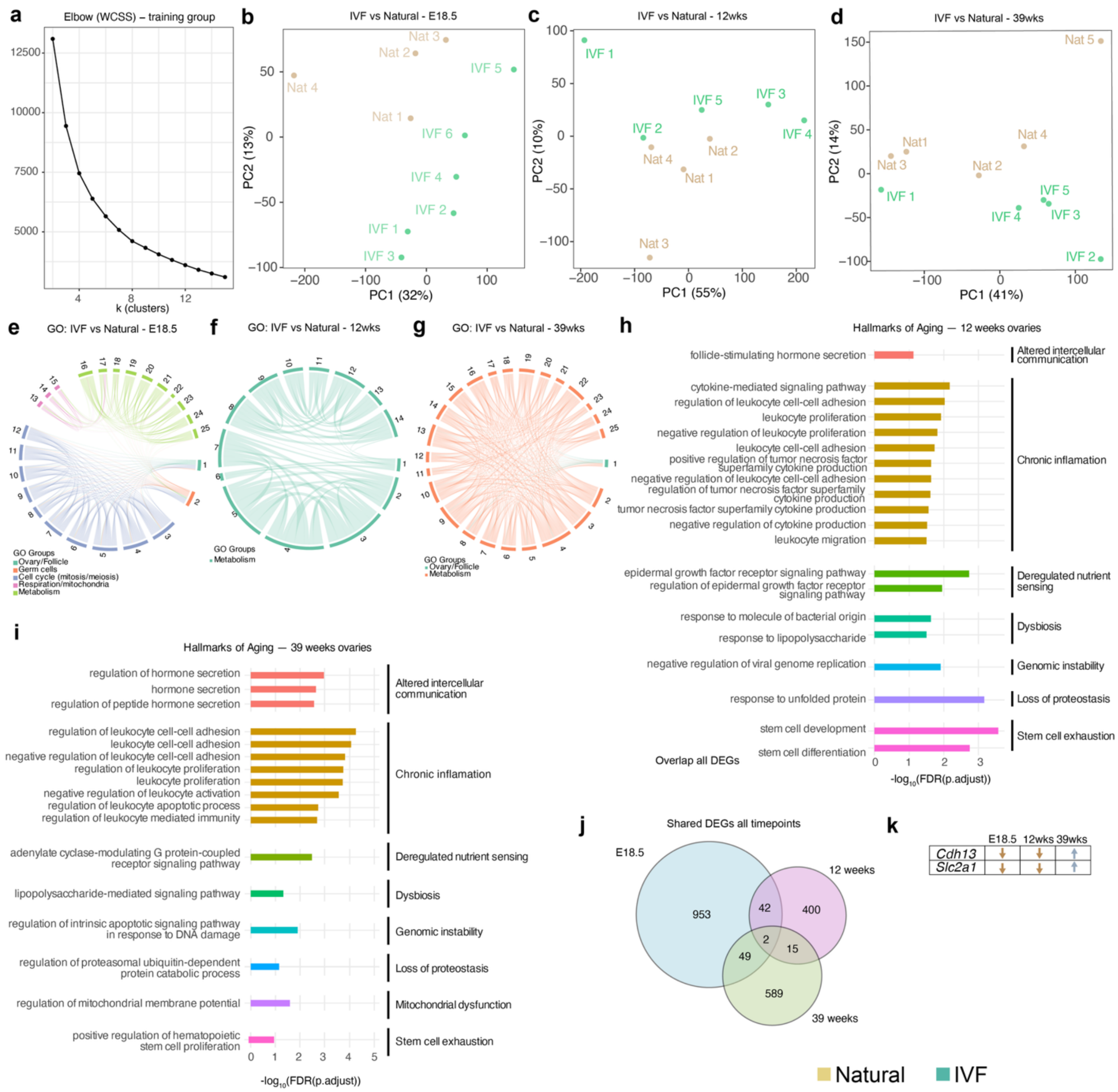

Extended Data 1. Corresponding to main Figure 3.

a, Elbow: plot analysis for k selection in WCSS analysis. Principal Component Analysis (PCA) for whole ovary RNA-seq in Figure 3: **b,** E18.5; **c,** 12-weeks; **d,** 39-weeks. Chord plots for Network representation of most affected GO terms at **e,** E18.5; **f,** 12-weeks; **g,** 39-weeks. **h,** Venn diagram to find the overlap genes at the three developmental timepoints. **i,** List of genes that are shared between the different timepoints and the direction of their dysregulation.

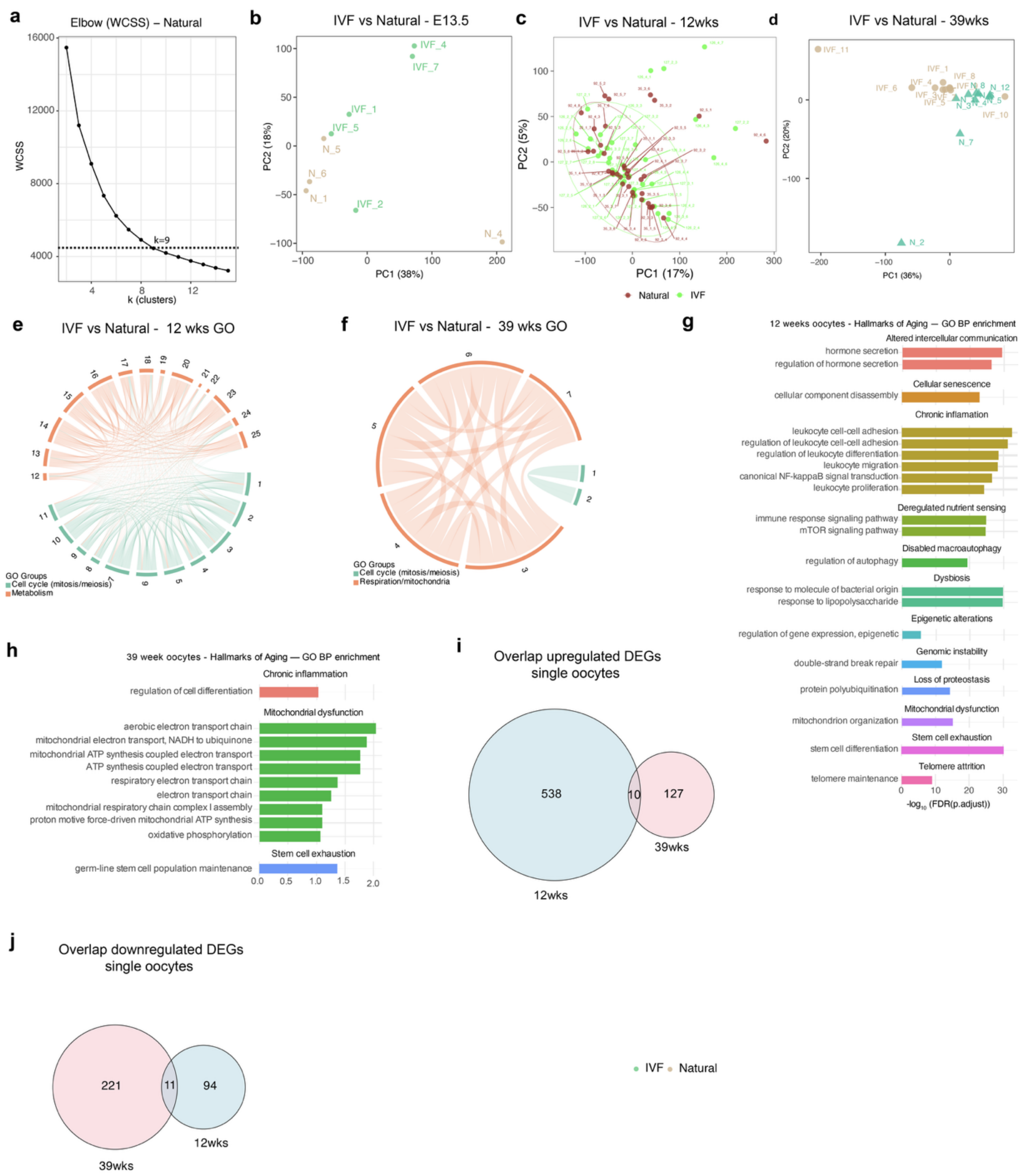

Extended Data 2. Corresponding to main Figure 4.

a, Elbow: plot analysis for k selection in WCSS analysis. Principal Component Analysis (PCA) for primordial germ cells and oocytes RNA-seq in Figure 4: **b,** E13.5; **c,** 12-weeks; **d,** 39-weeks. Chord plots for Network representation of most affected GO terms at **e,** 12-weeks; **f,** 39-weeks. Hallmarks of aging: **g,** 12-weeks; **h,** 39-weeks. Overlap between 12 and 39 weeks for **i,** upregulated DEGs and **j,** downregulated DEGs.

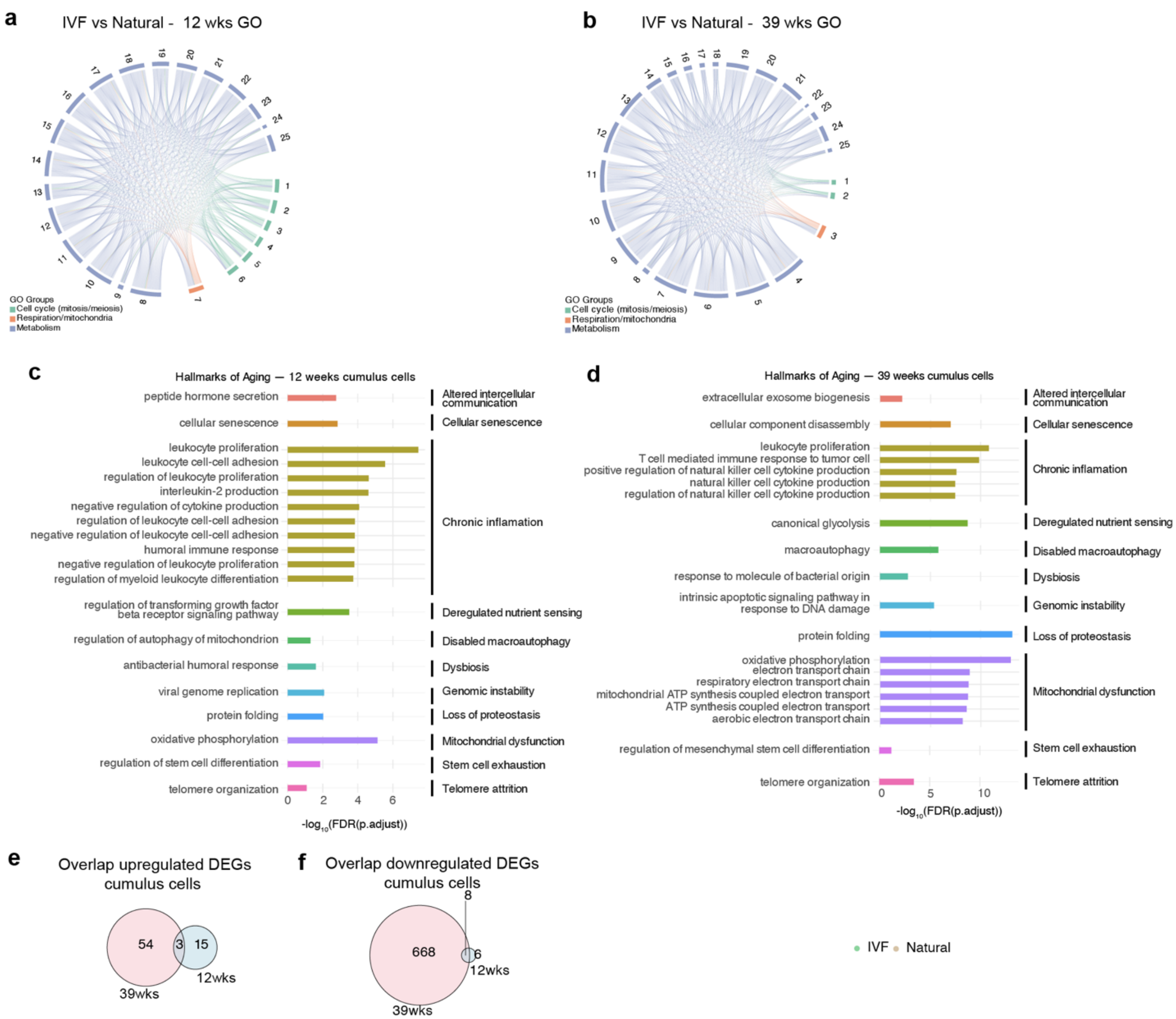

Extended Data 3. Corresponding to main Figure 5.

Principal Component Analysis (PCA) for cumulus cells RNA-seq in Figure 4: **a,** 12 weeks; **b,** 39 weeks. Chord plots for Network representation of most affected GO terms at **c,** 12 weeks; **d,** 39 weeks. Gene ontology to identify hallmark of aging pathways based on López-Otín et al.2023: **e,** 12 weeks and **f,** 39 weeks. Overlap between 12 and 39 weeks for **g,** upregulated DEGs and **h,** downregulated DEGs.
