# Supplementary material for: Mouse offspring conceived by in vitro fertilization exhibit accelerated reproductive aging through premature ovarian insufficiency
Source: J Clin Invest. 2026 Jun 30;136(16):e201633. doi: 10.1172/JCI201633 (PMC13476136; doi:10.1172/JCI201633)
Supplement: Supplementary file 2 [file jci-136-201633-s143.pdf]

Gel 1

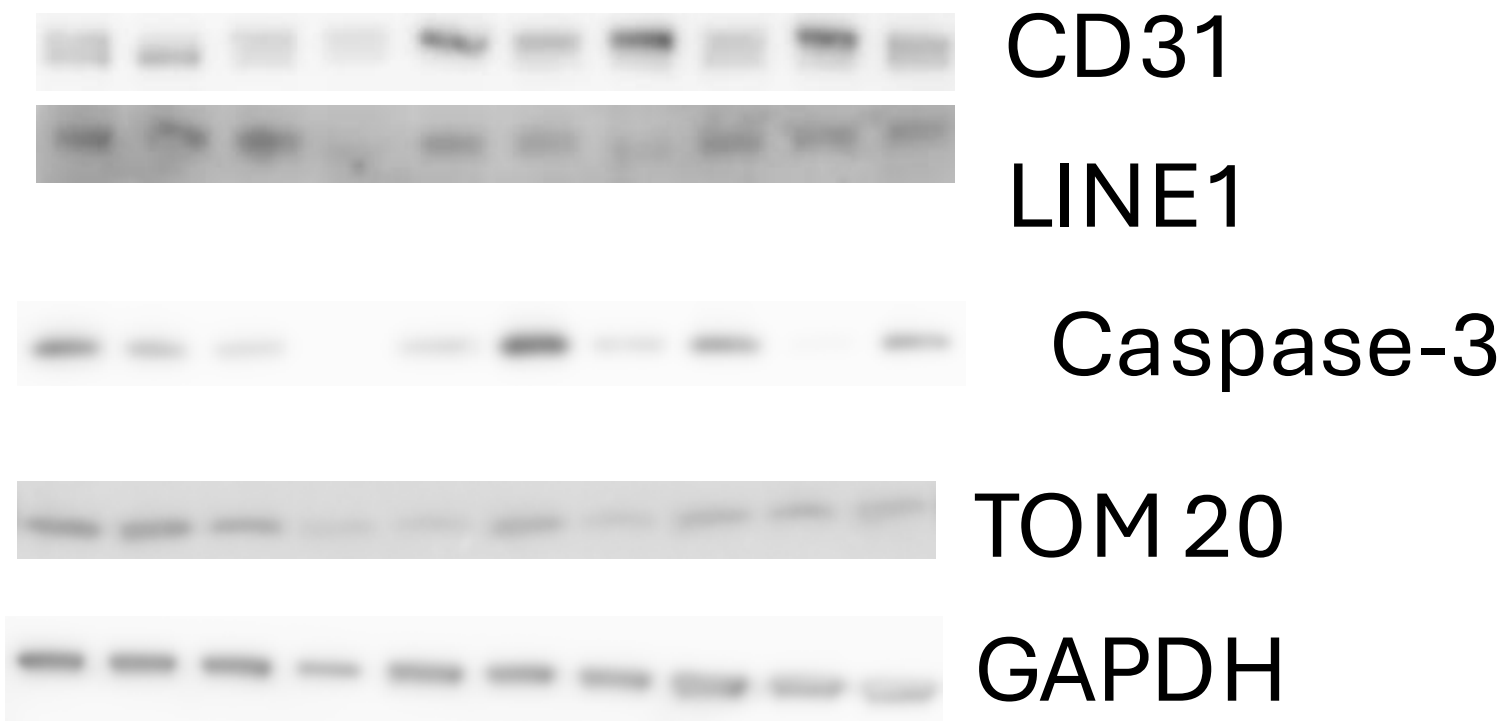

CD31

Gel 1

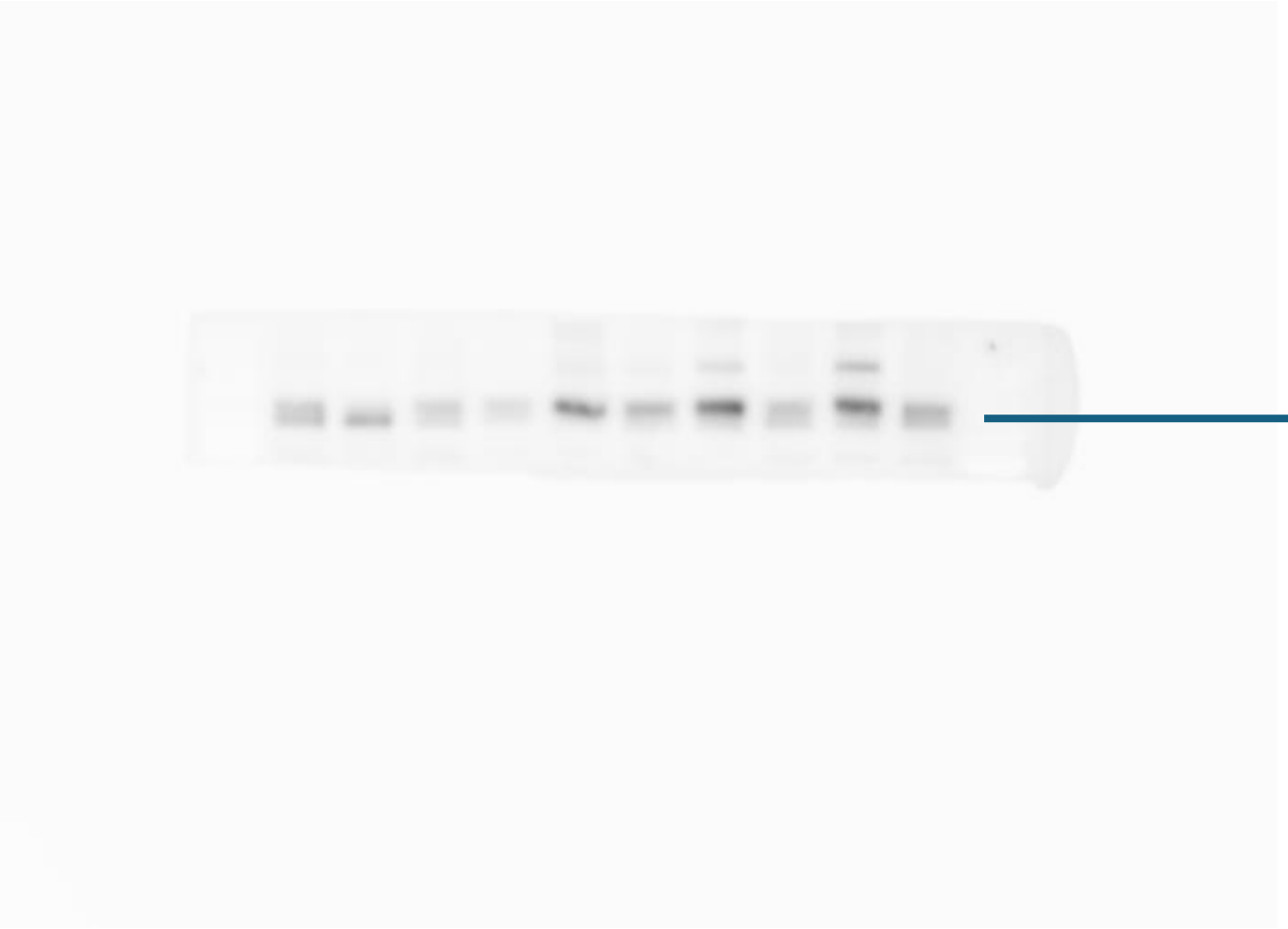

CD31

Gel 2

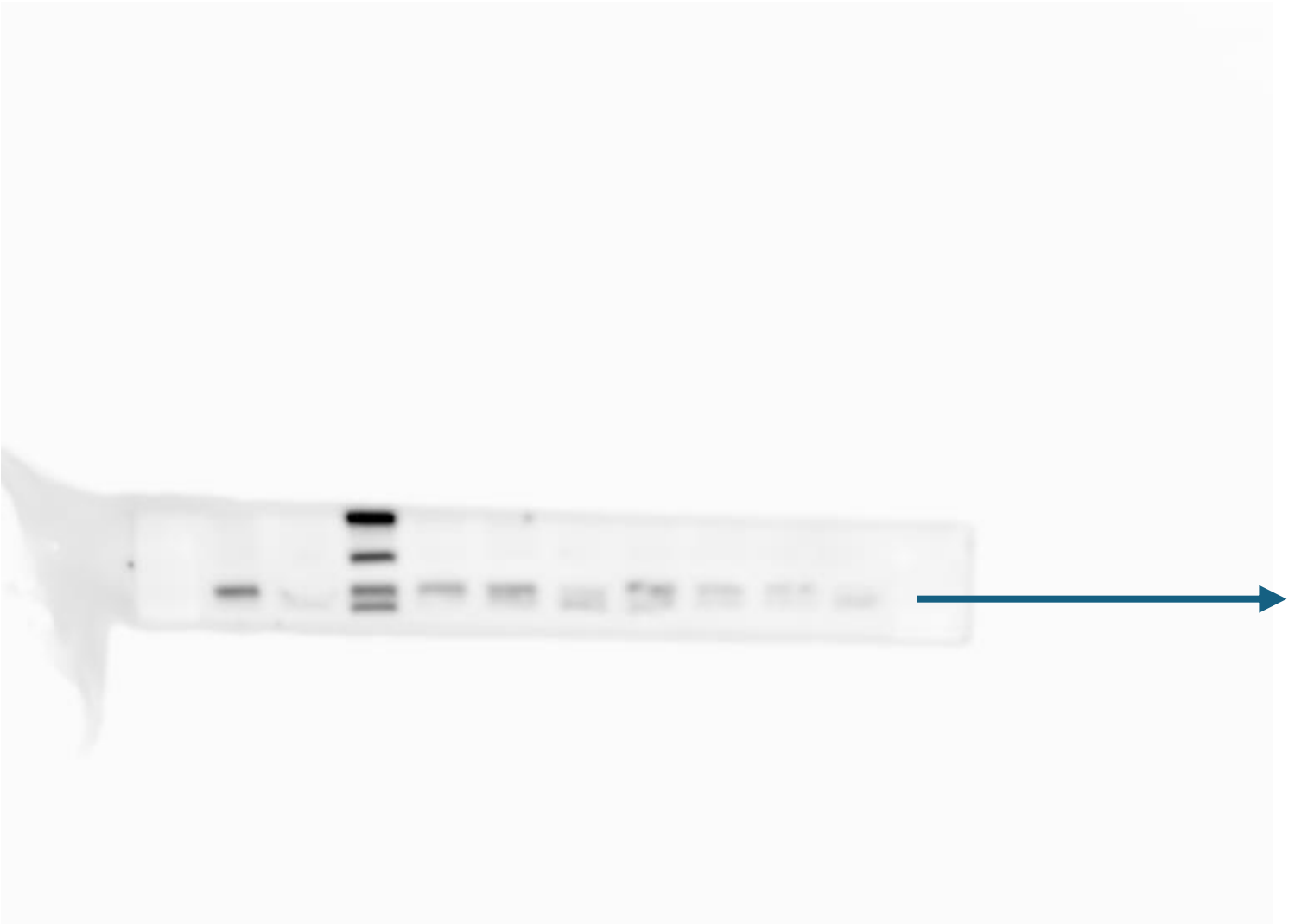

CD31

Cleaved Caspase 3

Gel 1

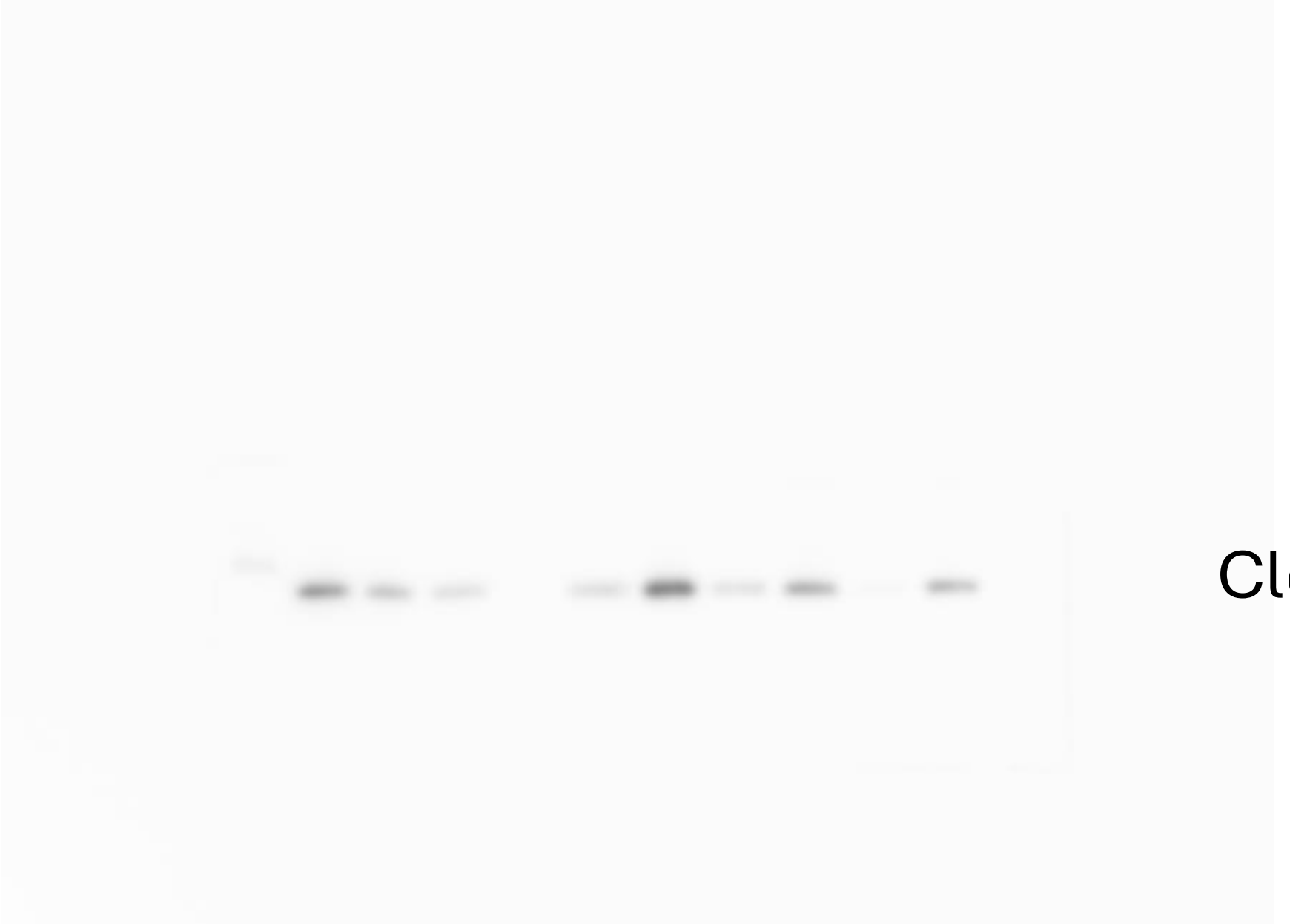

Cleaved Caspase 3

Gel 2

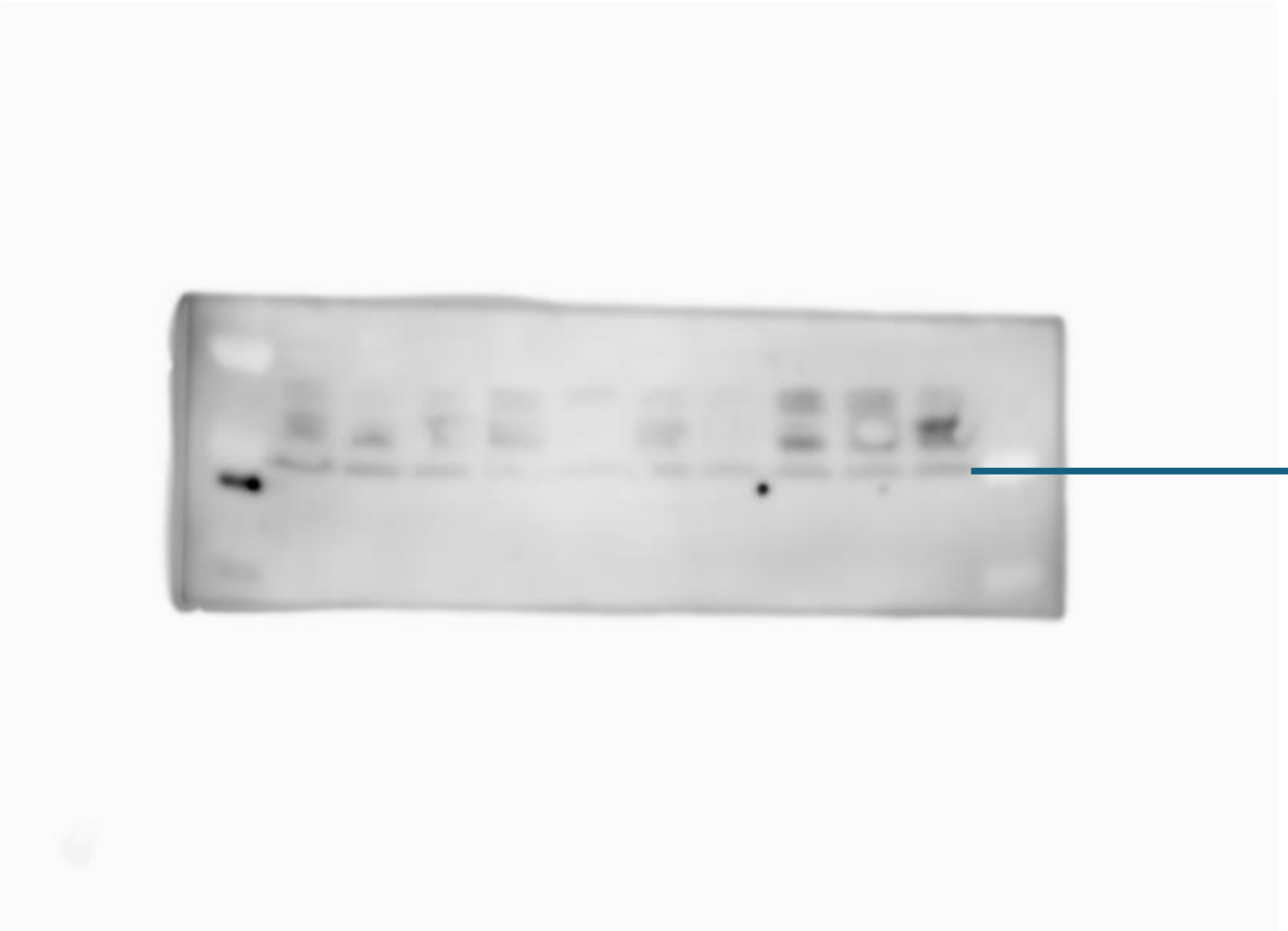

Cleaved Caspase 3

LINE1

Gel 1

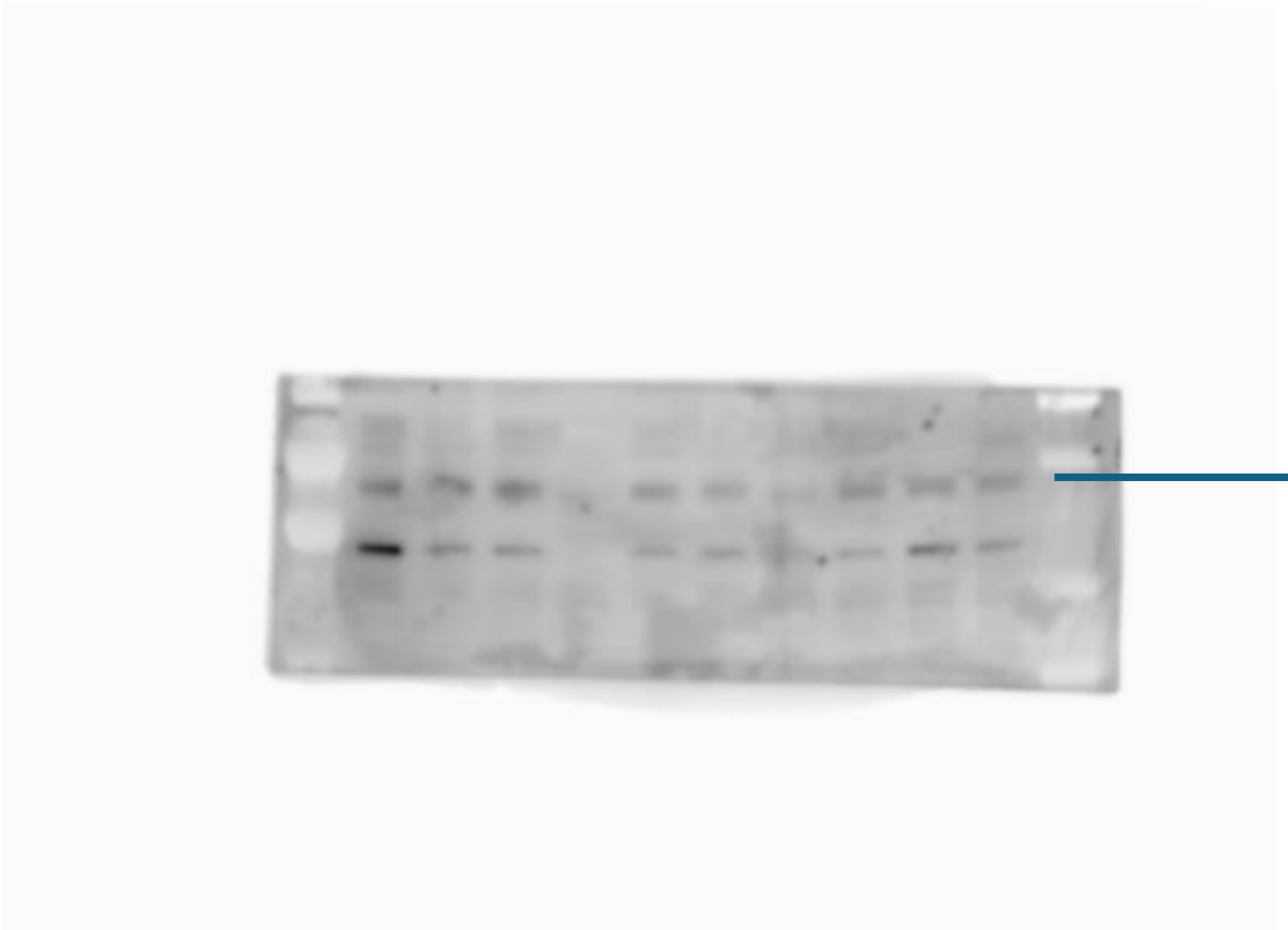

LINE1

Gel 2

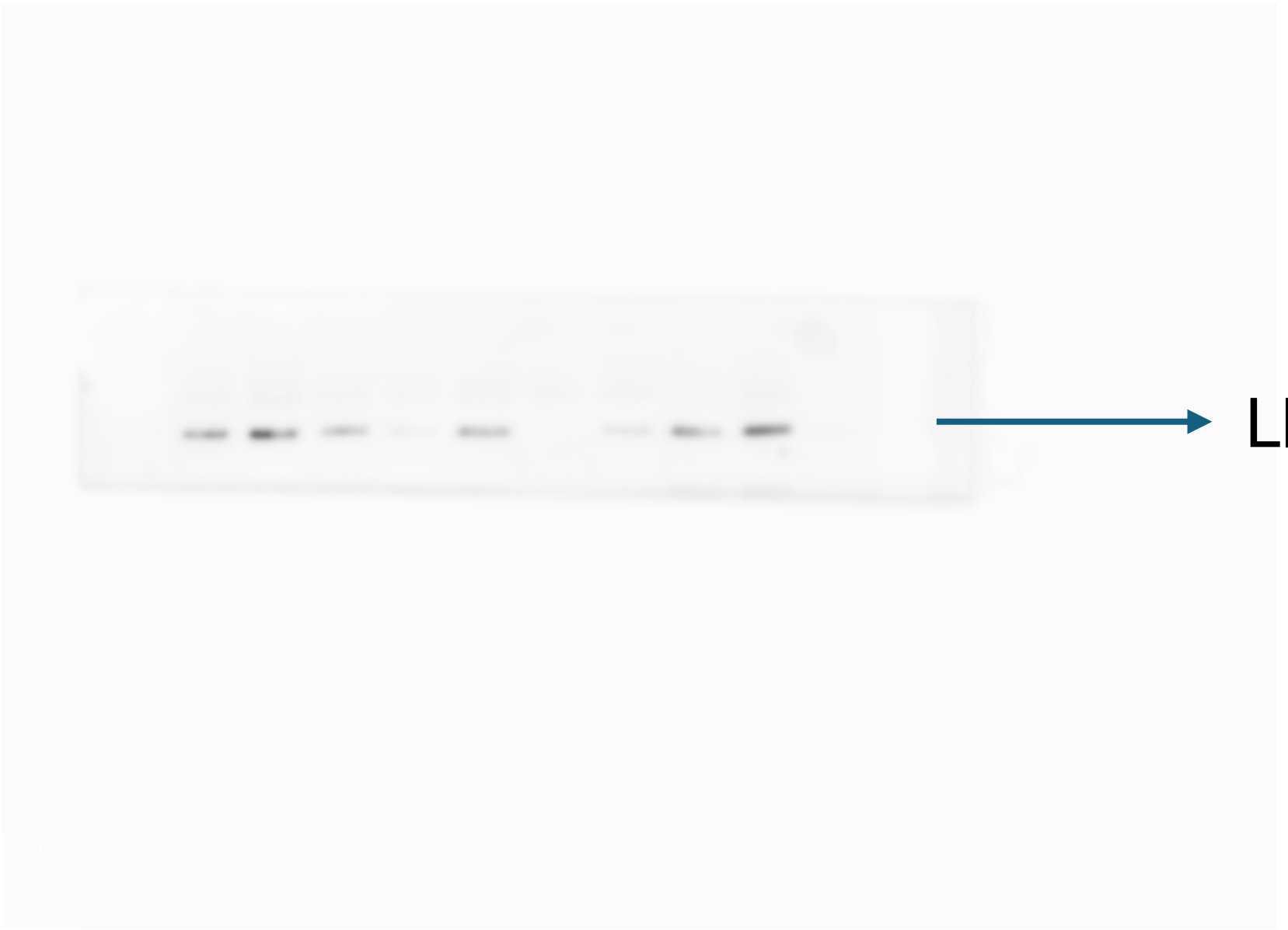

LINE1

TOMM20

Gel 1

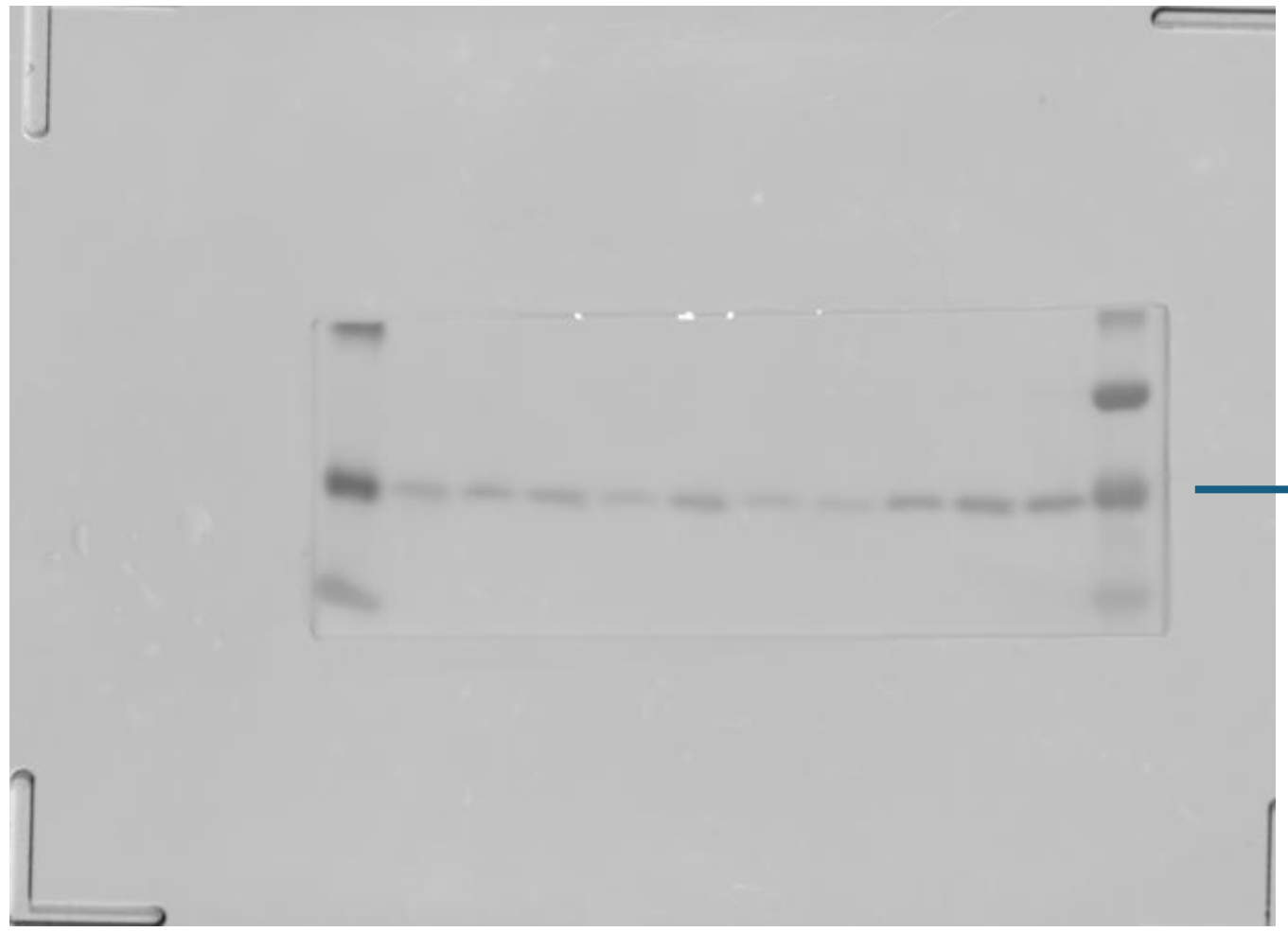

TOMM20

Gel 2

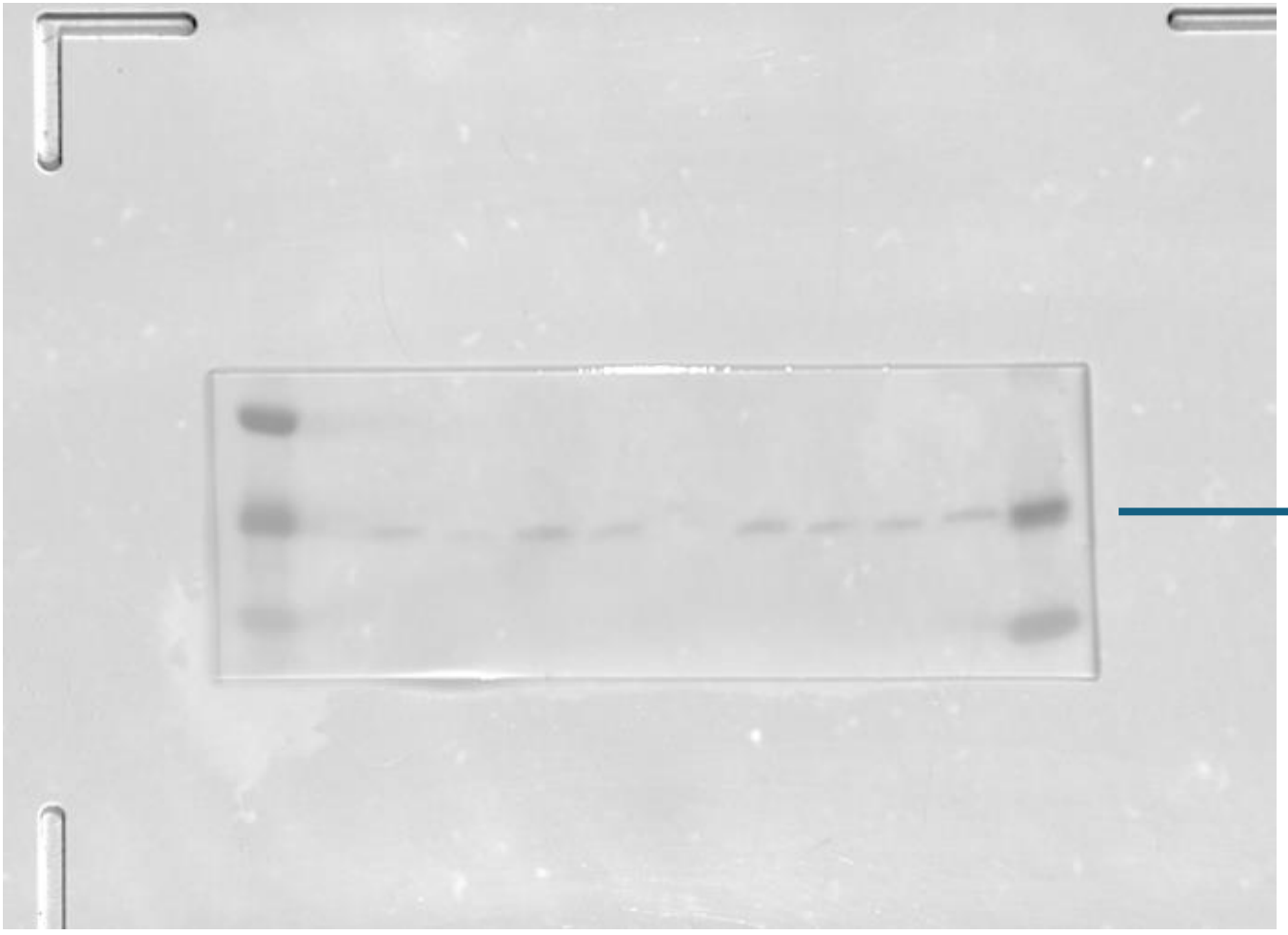

TOMM20

Gel 3

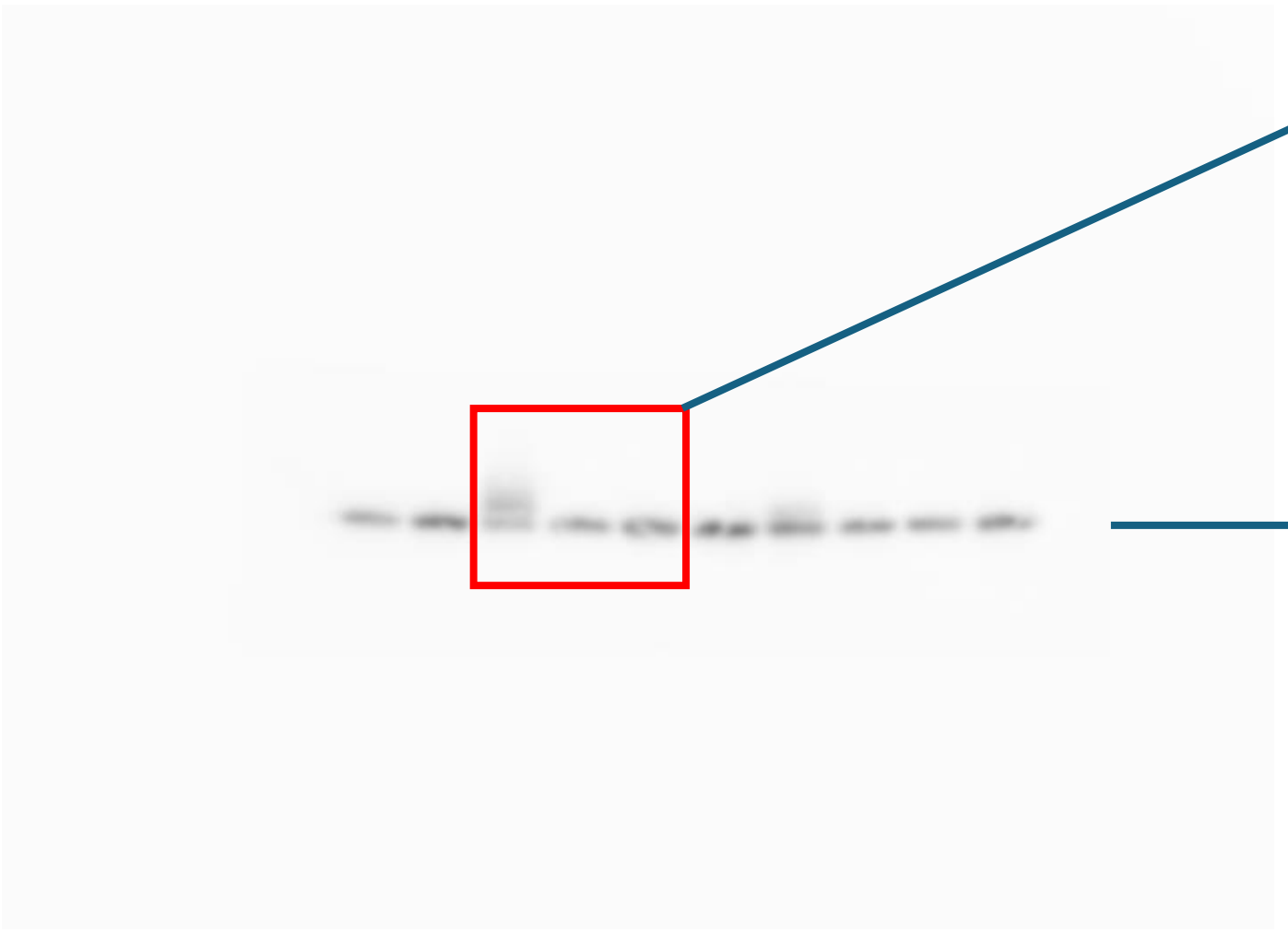

Not used for quantification since GAPDH band was damaged

TOMM20

GAPDH

Gel 1

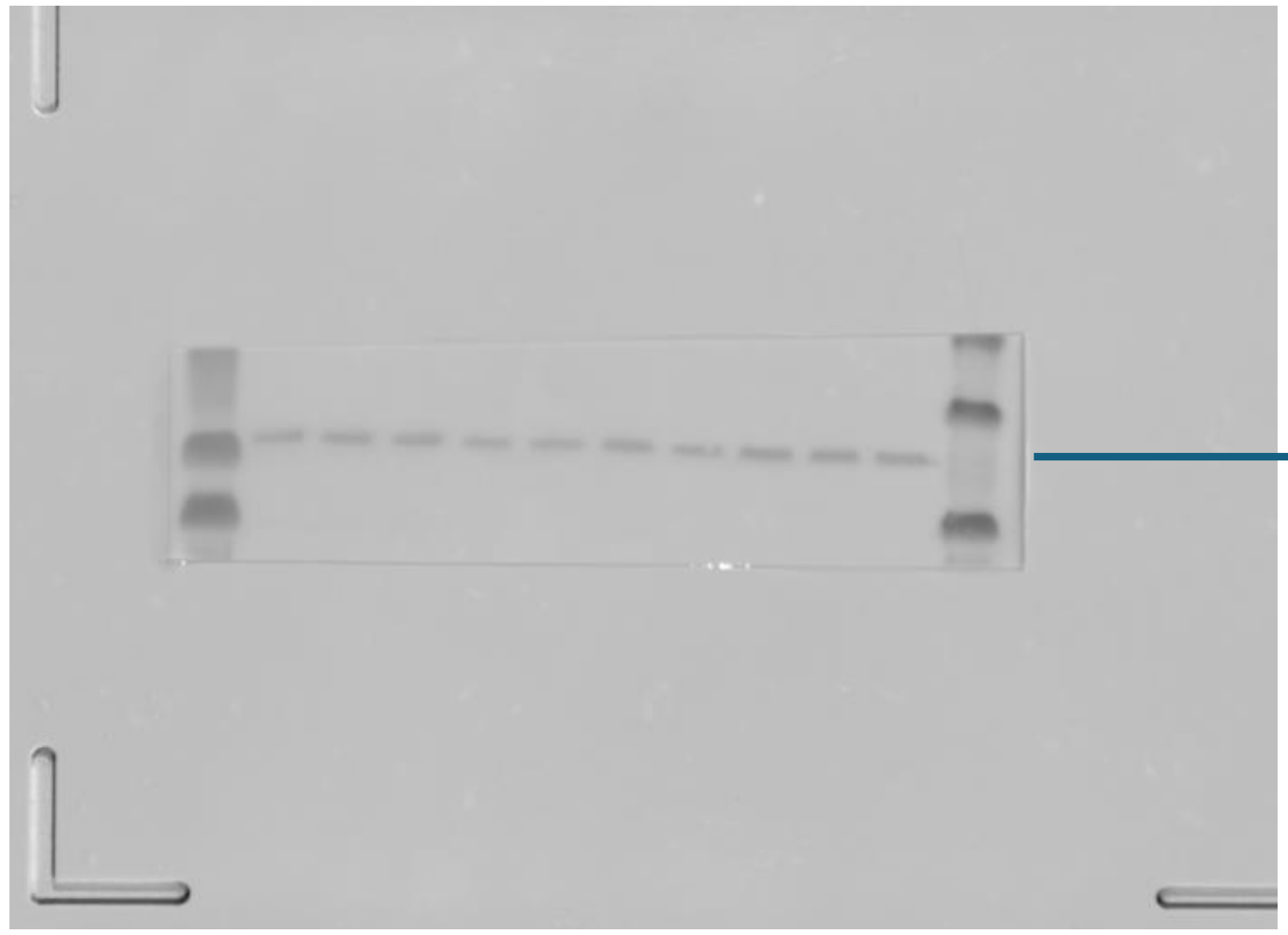

GAPDH

Gel 2

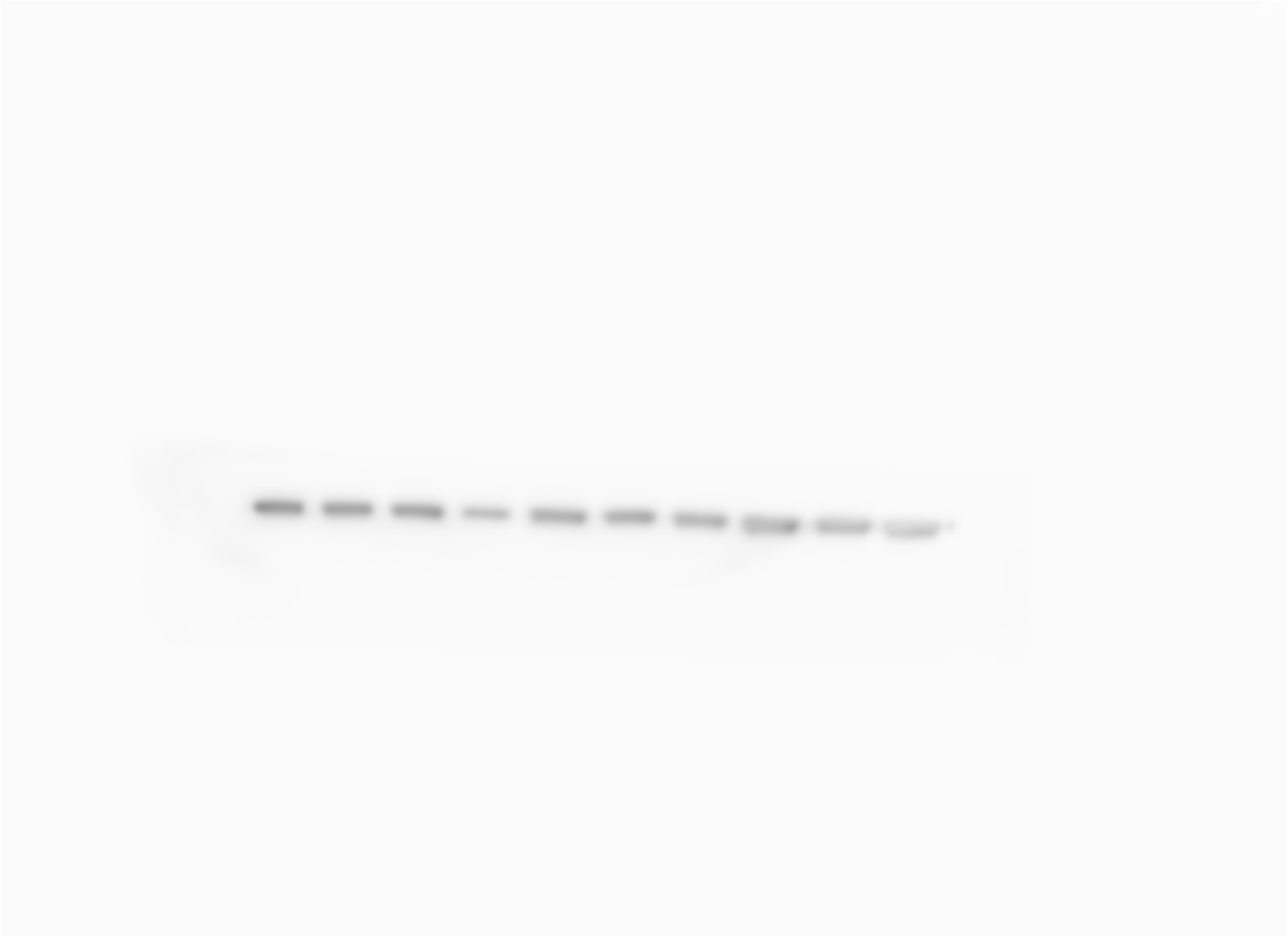

GAPDH

Gel 3

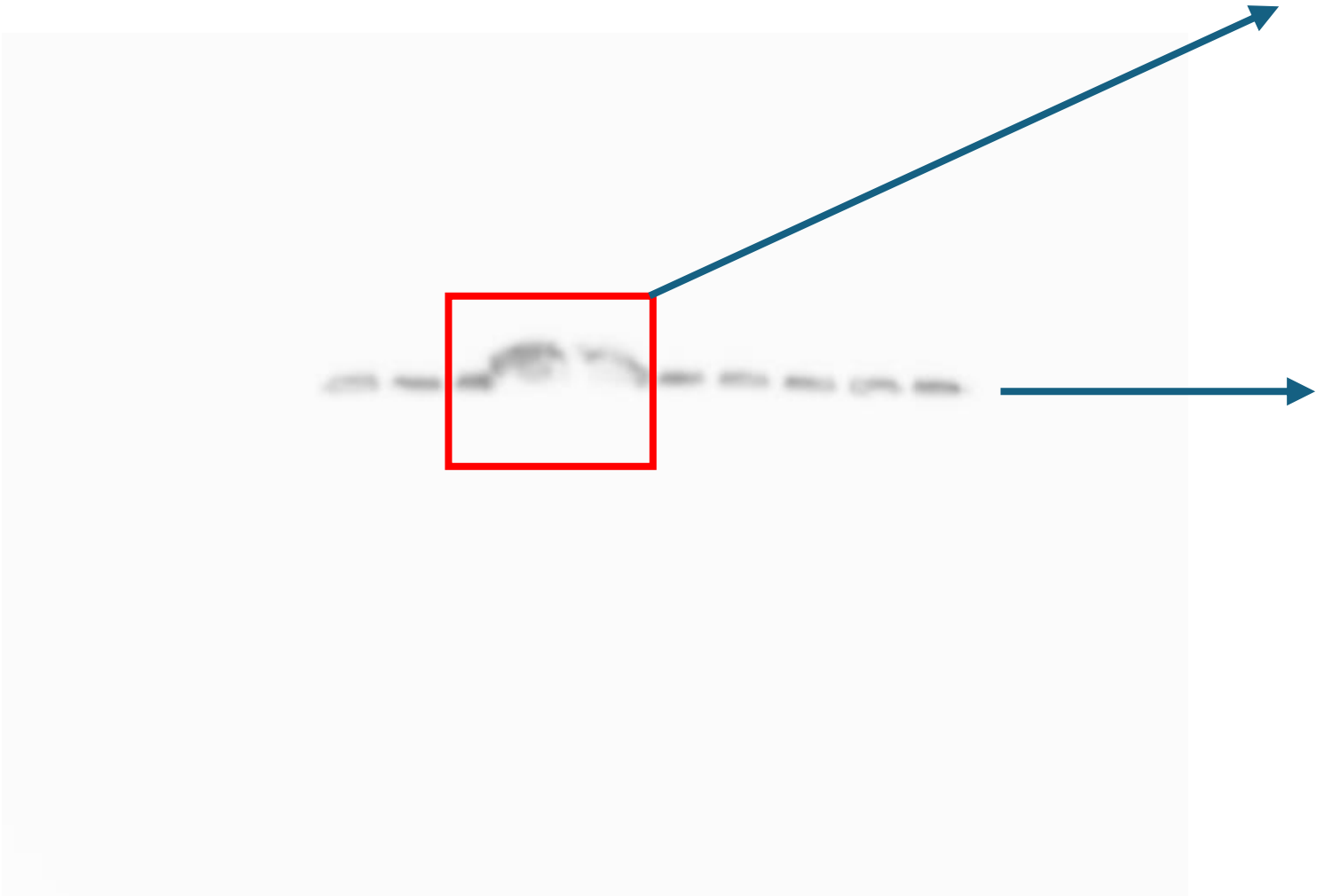

Not used for quantification

GAPDH
